# Multi-modal characterisation of early-stage, subclinical cardiac deterioration in patients with type 2 diabetes

**DOI:** 10.1186/s12933-024-02465-y

**Published:** 2024-10-19

**Authors:** Ambre Bertrand, Andrew Lewis, Julia Camps, Vicente Grau, Blanca Rodriguez

**Affiliations:** 1 Computational Cardiovascular Science Group, Department of Computer Science, University of Oxford, Oxford, OX1 3QD UK; 2 Division of Cardiovascular Medicine, Radcliffe Department of Medicine, University of Oxford, Oxford, OX3 9DU UK; 3 Institute of Biomedical Engineering, Department of Engineering Science, University of Oxford, Oxford, OX3 7DQ UK

**Keywords:** Diabetes mellitus (type 2), Cardiovascular diseases, Electrocardiography, Magnetic resonance imaging, Cross-sectional studies, UK Biobank

## Abstract

**Background:**

Type 2 diabetes mellitus (T2DM) is a major risk factor for heart failure with preserved ejection fraction and cardiac arrhythmias. Precursors of these complications, such as diabetic cardiomyopathy, remain incompletely understood and underdiagnosed. Detection of early signs of cardiac deterioration in T2DM patients is critical for prevention. Our goal is to quantify T2DM-driven abnormalities in ECG and cardiac imaging biomarkers leading to cardiovascular disease.

**Methods:**

We quantified ECG and cardiac magnetic resonance imaging biomarkers in two matched cohorts of 1781 UK Biobank participants, with and without T2DM, and no diagnosed cardiovascular disease at the time of assessment. We performed a pair-matched cross-sectional study to compare cardiac biomarkers in both cohorts, and examined the association between T2DM and these biomarkers. We built multivariate multiple linear regression models sequentially adjusted for socio-demographic, lifestyle, and clinical covariates.

**Results:**

Participants with T2DM had a higher resting heart rate (66 vs. 61 beats per minute, *p* < 0.001), longer QTc interval (424 vs. 420ms, *p* < 0.001), reduced T wave amplitude (0.33 vs. 0.37mV, *p* < 0.001), lower stroke volume (72 vs. 78ml, *p* < 0.001) and thicker left ventricular wall (6.1 vs. 5.9mm, *p* < 0.001) despite a decreased Sokolow-Lyon index (19.1 vs. 20.2mm, *p* < 0.001). T2DM was independently associated with higher heart rate (beta = 3.11, 95% CI = [2.11,4.10], *p* < 0.001), lower stroke volume (beta = −4.11, 95% CI = [−6.03, −2.19], *p* < 0.001) and higher left ventricular wall thickness (beta = 0.133, 95% CI = [0.081,0.186], *p* < 0.001). Trends were consistent in subgroups of different sex, age and body mass index. Fewer significant differences were observed in participants of non-white ethnic background. QRS duration and Sokolow-Lyon index showed a positive association with the development of cardiovascular disease in cohorts with and without T2DM, respectively. A higher left ventricular mass and wall thickness were associated with cardiovascular outcomes in both groups.

**Conclusion:**

T2DM prior to cardiovascular disease was linked with a higher heart rate, QTc prolongation, T wave amplitude reduction, as well as lower stroke volume and increased left ventricular wall thickness. Increased QRS duration and left ventricular wall thickness and mass were most strongly associated with future cardiovascular disease. Although subclinical, these changes may indicate the presence of autonomic dysfunction and diabetic cardiomyopathy.

**Graphical Abstract:**

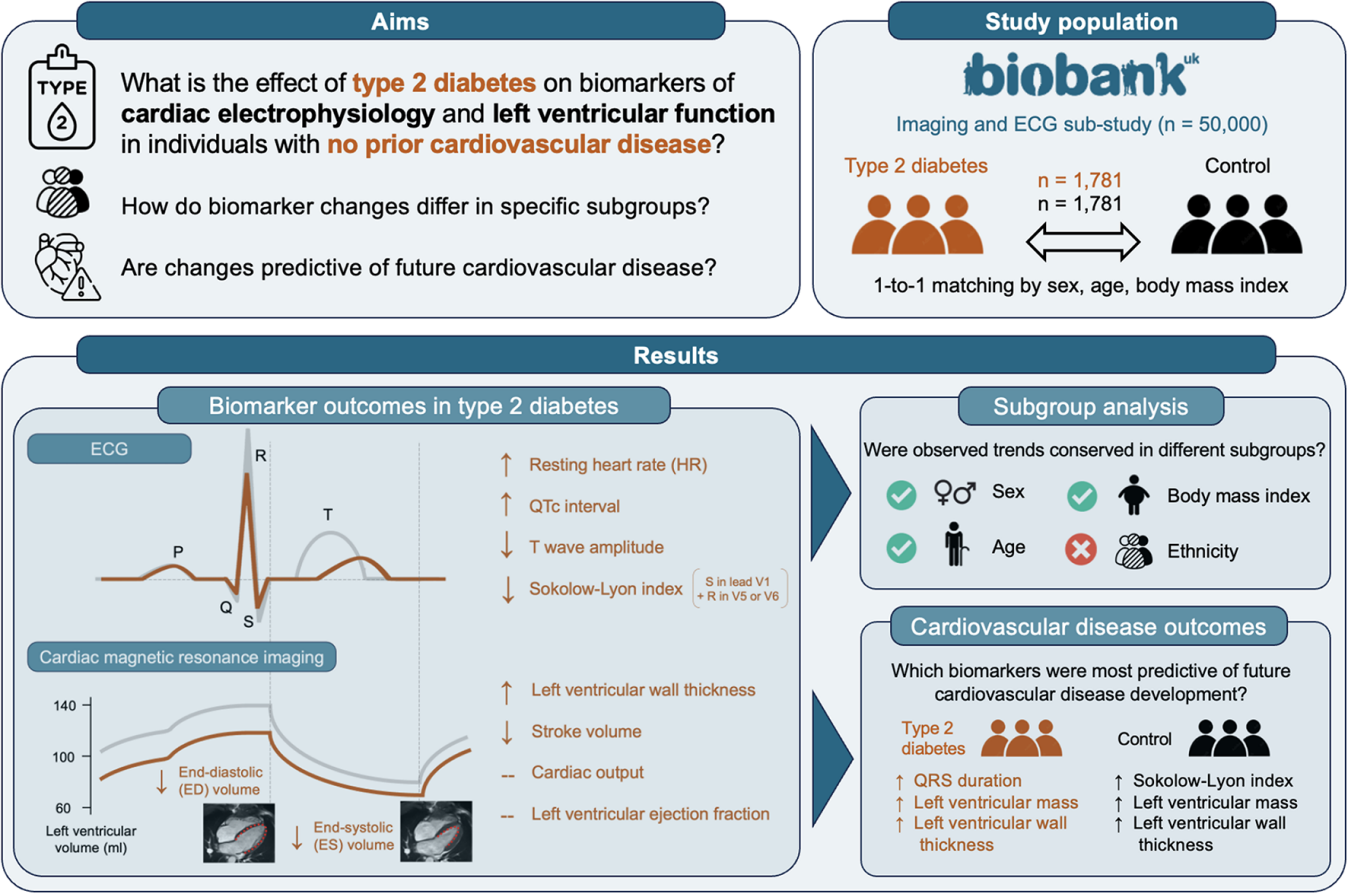

**Supplementary Information:**

The online version contains supplementary material available at 10.1186/s12933-024-02465-y.

## Introduction

Type 2 diabetes mellitus (T2DM) is a complex metabolic disease that affects over 536 million adults worldwide, according to the International Diabetes Federation (IDF) [1]. T2DM causes a two- to four-fold increase in risk of cardiovascular disease (CVD), the leading cause of death globally, which claims the lives of 17.9 million people annually according to the World Health Organisation [2, 3]. Thus, there is a pressing need to better understand the interplay between T2DM and the cardiovascular system, especially the identification of early signs of cardiac deterioration in T2DM, in order to mitigate disease progression and ultimately reduce deaths.

T2DM is characterised by chronically elevated blood glucose levels, known as hyperglycaemia, due to insulin resistance. Hyperglycaemia triggers a series of adverse molecular changes that lead to myocardial fibrosis, stiffness, and contractile dysfunction. These changes worsen progressively over time, eventually resulting in diastolic dysfunction and heart failure if left uncontrolled. In the absence of cardiovascular disease, these functional changes in T2DM have been referred to as diabetic cardiomyopathy or diabetic myocardial disorder, however this concept is yet to be confirmed according to the European Society of Cardiology [2, 4, 5]. Additionally, diabetes is associated with electrophysiological changes caused by the modulation of cardiac ionic currents, alterations in gap junctions, and abnormal conduction due to fibrosis [6–8]. Cardiac autonomic neuropathy (CAN), a common yet underdiagnosed complication of diabetes, is also responsible for altering electrophysiological function and heart rate due to damaged cardiac innervation [9]. Cardiac magnetic resonance (CMR) imaging studies have identified structural and functional changes in small cohorts of individuals with diabetes (n = 46, n = 143) [10, 11], while electrophysiological abnormalities in diabetes may manifest in the electrocardiogram (ECG), notably in the QT interval and T wave [12–15].

However, to our knowledge, no previous work has quantified ECG and CMR-derived biomarkers concurrently in a large, matched cohort of individuals with diabetes of type 2 specifically, before a clinical diagnosis of CVD. The goal of this study is to improve our understanding and the identification of subclinical T2DM-driven cardiac remodelling at a population level, and to assess the predictive value of multi-modal biomarkers in predicting CVD; the overarching aim being to support CVD risk stratification in patients with T2DM. We hypothesise that, compared to matched controls, biomarker differences in individuals with T2DM may provide further evidence pointing towards the existence of diabetic cardiomyopathy and electrophysiological abnormalities. Using the UK Biobank, we investigated the effect of T2DM on ECG and CMR-derived biomarkers reflecting cardiac morphology, haemodynamic function and electrophysiology, and quantify these biomarkers’ predictive value in relation to CVD development. Sex, age, body mass index, and ethnicity-related differences are investigated in subgroup analyses, as well as differences across the glycaemic spectrum.

## Methods

### Study design and population

The UK Biobank study is a multi-centre, prospective cohort study of over half a million adults recruited between the age of 40 and 69, and living in England, Scotland and Wales. It contains socio-demographic, lifestyle, and clinical information recorded at multiple timepoints since recruitment in 2006, and is linked to general practice primary care records and hospital episode statistics (HES).

Following a baseline assessment visit attended by all participants, about 50,000 participants selected at random were recalled for a multi-modal imaging assessment. This visit included a CMR scan performed on a 1.5 Tesla scanner (MAGNETOM Aera, Syngo Platform VD13A, Siemens Healthcare). Full details of the imaging protocol are available in [16]. A 12-lead resting ECG (CardioSoft ECG system, GE) was also recorded on the same day. Our primary analysis is a matched cross-sectional study examining the association between T2DM status and multi-modal cardiac biomarkers. As secondary analyses, we also carry out a case–control study quantifying the relationship between selected biomarkers in participants who did and did not develop CVD during follow-up, in two matched cohorts with and without T2DM.

Our study is reported in line with the STROBE (Strengthening the Reporting of Observational Studies in Epidemiology) statement.

### Ethical considerations

General ethical approval was granted for UK Biobank studies by the United Kingdom's National Health Service Research Ethics Service (11/NW/0382). Participants provided written informed consent for their data to be stored and used for research purposes. This study was conducted under UK Biobank Application Number 40161.

### Cohort definition

#### Disease ascertainment

**Fig. 1:**
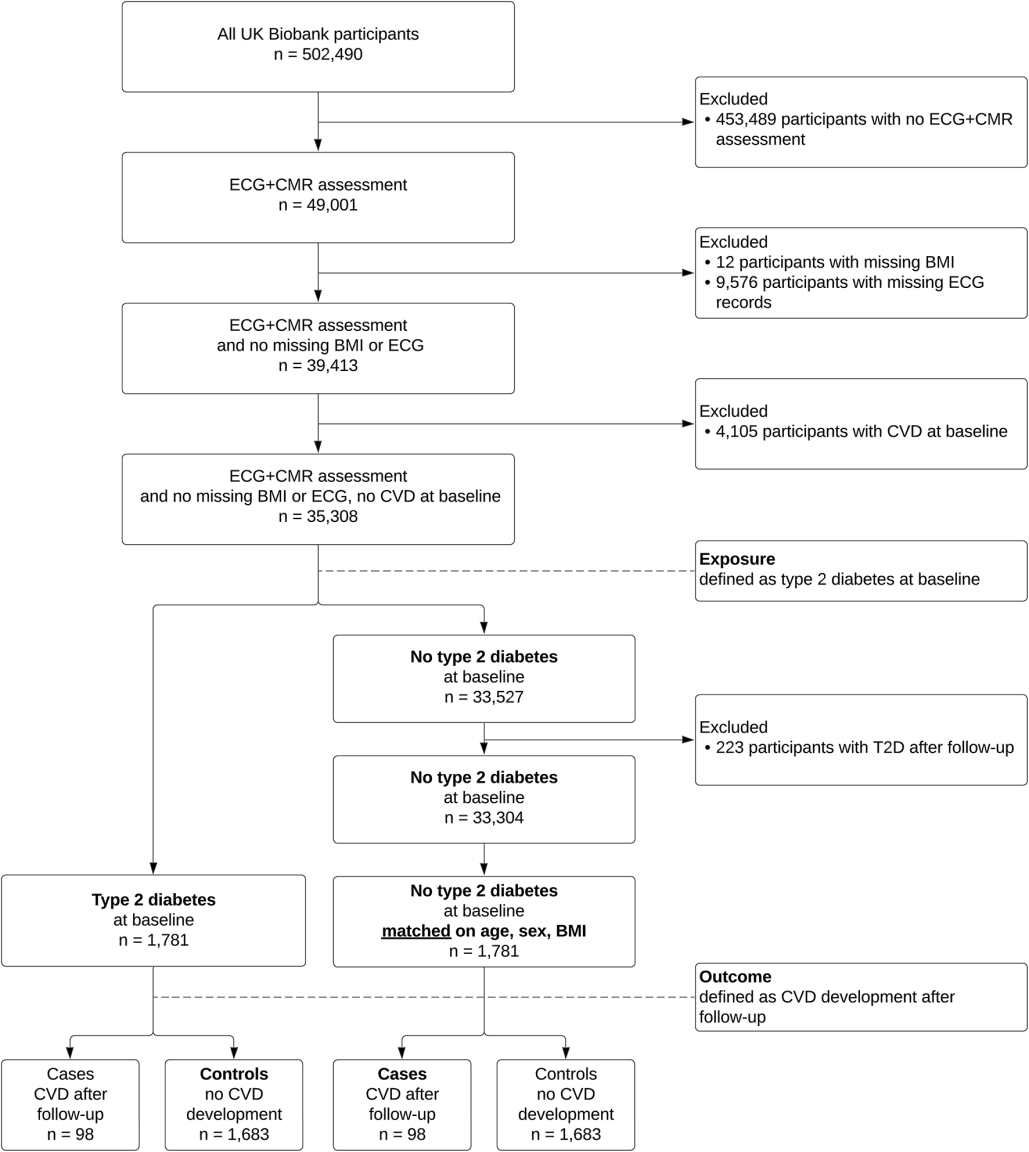
Cohort selection flowchart. CMR: cardiac magnetic resonance imaging, BMI: body mass index, CVD: cardiovascular disease, T2D: type 2 diabetes. Baseline refers to the time of recording of the ECG and CMR images. Follow-up refers to the period between baseline and censoring date (21/09/2021)

The selection process and cohort sizes are summarised in Fig. 1. We defined our cohorts based on the clinical ascertainment of two disease phenotypes, namely T2DM and CVD. Relevant ICD-9 and ICD-10 codes and their corresponding UK Biobank fields are listed in Supplementary Table 1. T2DM was determined by one or more of the following criteria: date of a T2DM ICD code first reported, HES record corresponding to a T2DM ICD code, HbA1c ≥ 48 mmol/mol, response to a patient touchscreen questionnaire [17]. CVD was determined by one or more of the following criteria: date of a CVD ICD code first reported, HES record corresponding to a CVD ICD code, date of myocardial infarction (MI), date of ST-elevated MI, and date of non-ST-elevated MI. The last three data fields are UK Biobank-specific algorithmically defined diagnoses of MI summarising information contained in other fields; cases identified via those fields may overlap with other routes of ascertainment but were included for completeness.

#### Exclusion criteria, exposure and outcomes

**Fig. 2:**
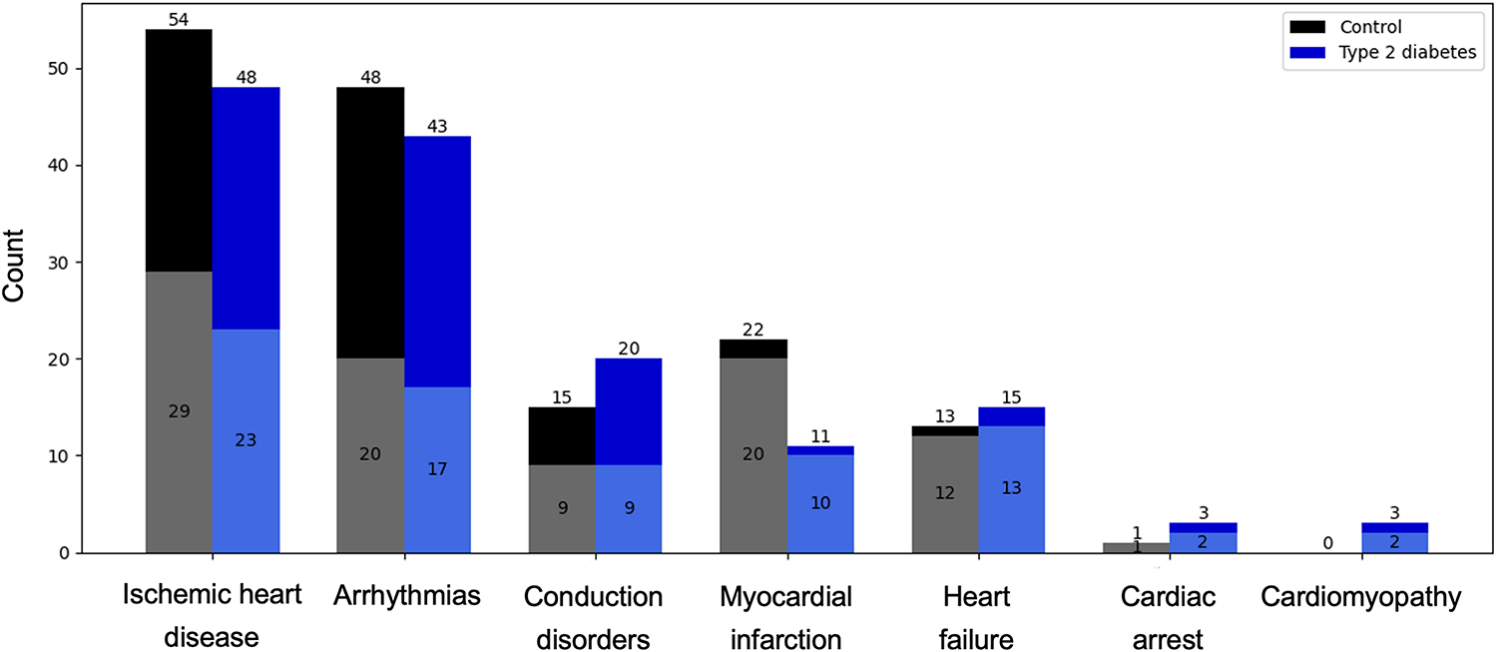
Incident cardiovascular disease (CVD) diagnoses in the control and type 2 diabetes cohorts during the follow-up period after the imaging and ECG assessment. Total count for each CVD category is indicated at the top of each bar. The shaded areas represent cases where this CVD exists in conjunction with one or more other CVD diagnoses. Each cohort includes n = 98 individuals with one or more CVD(s) developed during the follow-up period

We excluded all participants with pre-existing CVD, and censored all participants with CVD diagnosed after 21/09/2021, the date of the latest CVD diagnosis identified at the time of analysis. The median follow-up period between date of assessment and date of first CVD diagnosis is 1.5 years (min 8 days, max 5.9 years). The breakdown of follow-up CVDs in each cohort is presented in Fig. 2.

The exposure cohort consists of eligible participants with known T2DM at time of assessment. Pair-matching was carried out to select controls with no known T2DM based on age, sex, body mass index (BMI), as well as binary occurrence of CVD during follow-up (i.e. with vs. without a CVD diagnosis). We use BMI as a suitable matching variable as it is a commonly used marker of adiposity that modulates the ECG [18]. Euclidian distances were computed between the [age, BMI] vectors of each exposure participant and possible control participants. Each exposure participant was matched by a control participant of the same sex and follow-up CVD status with the lowest [age, BMI] Euclidian distance [19].

We defined outcomes as ECG and CMR biomarker values for all analyses apart from a secondary analysis considering CVD development, where we defined an outcome as a case of CVD during follow-up.

### Baseline covariates

#### Demographic, lifestyle, and clinical characteristics

We included the following demographic, lifestyle, and clinical characteristics: age at assessment, sex, ethnicity, weight, height, smoking status, systolic blood pressure (SBP), diastolic blood pressure (DBP), medication. BMI was calculated using the ratio of weight in kilograms over squared height in meters. Information on medication was self-reported and divided into broad categories and/or using UK Biobank-specific individual medication codes recorded by nurses. We included cholesterol-lowering medication, blood pressure medication, insulin, metformin and rosiglitazone 1mg/metformin 500mg tablet. The following blood biochemistry measurements were included: high-density lipoprotein (HDL) cholesterol, total cholesterol, HbA1c, triglycerides, creatinine, C-reactive protein. These measurements were taken prior to the imaging assessment so we used the most recent one available. Estimated glomerular filtration rate (eGFR) was calculated using serum creatinine according to the CKD-EPI creatinine equation adjusted for age, sex and ethnicity [20]. Creatinine was converted to mg/dL by multiplying the given value in µmol/L by a factor of 0.0113 [21].

#### ECG and CMR-derived biomarkers

Resting ECG recordings were available in.xml file format. Each file included the raw ECG signal as well as automatically extracted key ECG markers. Wave amplitudes were available for all 12 leads, while other markers were available for lead I only. We used the following markers: ventricular rate, QRS duration, QTc interval, T wave offset, R-wave amplitude, S-wave amplitude, J point amplitude (equivalent to ST segment elevation or depression), and T wave amplitude. Pairwise Pearson correlation coefficients were calculated to assess inter-covariate correlations among T wave and J point amplitudes in different ECG leads (Supplementary Fig. 1). Two representative leads were retained, one limb lead (aVL) and one precordial lead (V3). We computed the Sokolow-Lyon index for left ventricular hypertrophy as the sum of the S amplitude in V1 and the maximum value of R amplitude in V5 or V6 [22].

Some key CMR-derived biomarkers were directly available in the UK Biobank database. Manual analyses, algorithms and validation of these biomarkers are described in detail in [23, 24]. We used the following in our study: left ventricular ejection fraction (LVEF), LV end-diastolic volume, LV end-systolic volume, LV stroke volume, cardiac output, LV myocardial mass, LV global average wall thickness. We calculated body surface area (BSA) using Du Bois’ formula, BSA = 0.007184 × *w*^0.425^ × *h*^0.725^ where *w* is weight in kilograms and *h* is height in cm, to additionally obtain LV mass index (LVMI = LV mass / BSA).

### Statistical analysis

All statistical analyses were performed using RStudio with R version 4.1.1.

Baseline covariates were compared between the exposure and control cohorts. Normality of continuous variable distributions was assessed using the Shapiro–Wilk test. The independent samples t-test was used for normal distributions and the Mann–Whitney U test was used for non-normal distributions. Missing data were not imputed. All statistical tests were two-sided. Statistical significance was defined as *p* < 0.05. Median, interquartile range (IQR) and missing measurements in the form of count and percentage of the full sample, are reported for each variable.

We used multivariate multiple linear regression to examine the association between T2DM and ECG and CMR biomarkers. A crude, unadjusted model (Model 0) was used to quantify the association of T2DM only with each biomarker. Three additional models were constructed incrementally to account for selected socio-demographic (Model 1), lifestyle (Model 2), and clinical covariates detailed in the previous section (Model 3; fully adjusted). In regression modelling, inter-correlation between predictor variables can sometimes dilute true associations and lead to larger, imprecise confidence intervals. To mitigate this effect, we calculated pairwise Pearson correlation coefficients between potential model covariates and, for each covariate pair with a coefficient magnitude greater than 0.3, one covariate was excluded and the other retained in the adjusted models. This 0.3 threshold has been used to assess covariate correlation in a previous study [25] and is also widely used in the field of medicine as an indicator of ‘fair’ correlation [26]. This determined the exclusion of the following covariates from our models: eGFR, HbA1c, systolic blood pressure, HDL cholesterol, cholesterol-lowering medication, and metformin (see Results, Table 3). Although HbA1c was excluded (Pearson correlation coefficient with T2DM = 0.57, Supplementary Fig. 2), a subgroup analysis was carried out to assess the association of HbA1c, as a proxy for hyperglycaemia, with biomarker outcomes of interest (see Subgroup analyses below). Models were fitted using the Ordinary Least Squares method, therefore scaling of variables was not required. Regression coefficients, their 95% confidence intervals, and *p*-values are reported for each outcome variable and model in the tables.

We compared biomarkers between cases who did and did not develop CVD, in both cohorts with and without T2DM. We assessed the association between biomarkers with significant differences, and CVD development. We built two sets of logistic regression models, one for each cohort, and examined how these associations differ in each cohort. We followed the same covariate processing and sequential adjustment approach as described previously.

### Subgroup analyses

We examined the role of sex, age, BMI and ethnicity by comparing biomarkers in stratified subgroups. We investigated the effect of severity of disease on selected biomarkers by considering HbA1c as a proxy for hyperglycaemia. We built regression models as described previously, this time using HbA1c as a continuous exposure variable, and biomarkers that were associated with T2DM as outcomes. This analysis was performed within the T2DM cohort only, to avoid a dichotomous HbA1c distribution due to lower HbA1c levels in the control cohort.

## Results

### Cohort description

Both the exposure and the control cohorts (T2DM vs. no T2DM) consisted of 1,781 subjects matched by age, sex and BMI. Cohorts were mostly male (n = 1133, 63.6%), had a median age of 67 years and a median BMI of 27.8 kg/m^2^, which is considered overweight by the NHS (NHS Digital, 2022) (Table 1). Individuals with T2DM were more likely to be non-white (7.9% vs. 2.7%) and to be current or previous smokers (43.5% vs. 40.6%). They tended to have lower diastolic blood pressure, total cholesterol, and HDL cholesterol, while HbA1c, triglycerides, C-reactive protein, and eGFR were generally more elevated. Subjects with T2DM were also more likely to be on cholesterol-lowering drugs, anti-hypertensives, insulin and metformin. One patient in the control cohort reported taking insulin and metformin. This may be due to a lack of diabetes diagnosis at the time of visit, or an increase in HbA1c levels after the last measurement, leading to a missed positive classification of disease. Although rare, this case could also be due to the medication being used to treat another condition unrelated to diabetes, such as polycystic ovary syndrome [27].

**Table 1:** Socio-demographic, lifestyle, and clinical characteristics recorded for the cohorts with and without type 2 diabetes

	No type 2 diabetes		Type 2 diabetes		t-statistic	*p*-value
		N (%)		N (%)		
Full cohort, n (%)	–	1781 (100)	–	1781 (100)	–	–
*Socio-demographics*						
Age, years (median (IQR))	67 (60–71)	1781 (100)	67 (60–71)	1781 (100)	–	0.63
Sex: female, n (%)	648 (36.4)	1781 (100)	648 (36.4)	1781 (100)	0	1
Ethnicity	–	–	–	–	66.8	< 0.001
White, n (%)	1733 (97.3)	1781 (100)	1640 (92.1)	1781 (100)	–	–
Black, n (%)	15 (0.8)	1781 (100)	37 (2.1)	1781 (100)	–	–
Other, n (%)	33 (1.9)	1781 (100)	104 (5.8)	1781 (100)	–	–
*Lifestyle characteristics*						
BMI, kg/m^2^ (median (IQR))	27.7 (24.8–31.2)	1781 (100)	28.0 (25.1–31.6)	1781 (100)	–	0.05
Smoking status	–	–	–	–	6.4	0.04
Never, n (%)	1047 (59.4)	1762 (99.0)	994 (56.4)	1761 (98.9)	–	–
Previous, n (%)	648 (36.8)	1762 (99.0)	694 (39.4)	1761 (98.9)	–	–
Current, n (%)	67 (3.8)	1762 (99.0)	73 (4.1)	1761 (98.9)	–	–
*Clinical characteristics*						
Average SBP, mmHg (median (IQR))	142 (131–155)	1577 (88.5)	142 (132–155)	1560 (87.6)	–	0.68
Average DBP, mmHg (median (IQR))	81 (74–88)	1577 (88.5)	79 (72–85)	1560 (87.6)	–	< 0.001
Total cholesterol, mmol/L (median (IQR))	5.7 (5.0–6.5)	1673 (93.9)	5.1 (4.2–6.0)	1686 (94.7)	–	< 0.001
HDL cholesterol, mmol/L (median (IQR))	1.4 (1.1–1.6)	1557 (87.4)	1.2 (1.0–1.4)	1580 (88.7)	–	< 0.001
HbA1c, mmol/mol (median (IQR))	34.7 (32.5–37.1)	1669 (93.7)	43.7 (38.9–51.0)	1672 (93.9)	–	0
Creatinine, µmol/L (median (IQR))	75.6 (65.5–84.4)	1672 (93.9)	73.5 (63.5–83.4)	1684 (94.6)	–	< 0.001
Triglycerides, mmol/L (median (IQR))	1.6 (1.1–2.2)	1672 (93.9)	1.9 (1.3–2.7)	1681 (94.4)	–	< 0.001
C-reactive protein, mg/L (median (IQR))	1.4 (0.7–2.8)	1673 (93.9)	1.8 (0.9–3.5)	1681 (94.4	–	< 0.001
eGFR, ml/min/1.73m^2^ (median (IQR))	86.1 (77.5–92.5)	1672 (93.9)	87.8 (78.2–94.3)	1684 (94.6)	–	< 0.001
Medication						
Cholesterol-lowering, n (%)	485 (27.2)	1781 (100)	1287 (72.3)	1781 (100)	1801.8	0
Antihypertensive, n (%)	487 (27.3)	1781 (100)	1003 (56.3)	1781 (100)	607.7	< 0.001
Insulin, n (%)	1 (0.1)	1781 (100)	244 (13.7)	1781 (100)	280.4	< 0.001
Metformin, n (%)	1 (0.1)	1781 (100)	945 (53.1)	1781 (100)	2009	0

IQR, inter-quartile range; BMI, body mass index; SBP, systolic blood pressure; DBP, diastolic blood pressure; HDL, high-density lipoprotein; HbA1c, glycated haemoglobin A1c; eGFR, estimated glomerular filtration rate. The t-statistic is reported for categorical variables assessed using the Chi-Squared test. All continuous variables are distributed non-normally and compared using the Mann–Whitney U-test

### Type 2 diabetes is associated with a higher resting heart rate, longer QTc interval, reduced T wave amplitude and lower Sokolow-Lyon index

Compared to matched controls, ventricular rate in T2DM was higher (+ 5 bpm, median: 66 IQR: [59–74] vs. 61 [55–68] bpm) and T wave offset was earlier (− 12 ms, 842 [820–864] vs. 854 [834–874] ms) (Table 2). T2DM was strongly associated with both biomarkers in all models (Table 3). This consistency is expected, given the known inverse correlation between T wave offset and ventricular rate. QRS duration was shorter (− 2 ms, 86 [79–93] vs. 88 [82–96] ms) and QTc interval was longer (+ 4 ms, 424 [408–440] vs. 420 [405–436] ms) in the T2DM cohort (Table 2). There was a statistically significant association of T2DM with QTc interval in all models but the fully adjusted one (beta = 1.83, 95% CI = [0.13, 3.53], *p* = 0.035 before adjustment vs. beta = 0.86, 95% CI = [− 1.22, 2.95], *p* = 0.416 after adjustment). (Table 3). However, the association of T2DM with QRS duration was significant only in the two last models adjusted for lifestyle and clinical factors (Model 2 and 3, Table 3). This suggests that the importance of this variable may increase relative to other covariates included in the later models. There were statistically significant differences in almost all wave amplitude biomarkers between the cohorts (Table 2). T2DM was associated with a lower T wave amplitude in V3 in all models, and a lower T wave amplitude and less elevated J-point in aVL in all models but the fully adjusted one (Table 3). T2DM was associated with a lower Sokolow-Lyon index in all models but the fully adjusted one (Tables 2 and 3).

**Table 2:** ECG and CMR-derived biomarkers recorded for the cohorts with and without type 2 diabetes

	No type 2 diabetes		Type 2 diabetes		*p*-value
	Median (IQR)	N (%)	Median (IQR)	N (%)	
Full cohort	–	1781 (100)	–	1781 (100)	–
*ECG*					
Ventricular rate, bpm	61 (55–68)	1781 (100)	66 (59–74)	1781 (100)	< 0.001
QRS duration, ms	88 (82–96)	1781 (100)	86 (79–93)	1781 (100)	0.005
QTc interval, ms	420 (405–436)	1781 (100)	424 (408–440)	1781 (100)	< 0.001
T-wave offset, ms	854 (834–874)	1781 (100)	842 (820–864)	1781 (100)	< 0.001
T-wave amplitude (V3), mV	0.37 (0.23–0.54)	1766 (99.2)	0.33 (0.21–0.49)	1765 (99.1)	< 0.001
T-wave amplitude (aVL), mV	0.11 (0.05–0.17)	1718 (96.5)	0.10 (0.04–0.15)	1669 (93.7)	< 0.001
J-point amplitude (V3), mV	− 0.02 ((− 0.04)− 0.02)	1781 (100)	− 0.02 ((− 0.04)− 0.02)	1781 (100)	0.89
J-point amplitude (aVL), mV	0.01 ((− 0.01)− 0.03)	1781 (100)	0.00 ((− 0.02)− 0.03)	1781 (100)	0.016
Sokolow-Lyon index, mm	20.2 (16.2–24.8)	1650 (92.6)	19.1 (15.2–23.5)	1612 (90.5)	< 0.001
*CMR*					
LVEF, %	56 (52–59)	1533 (86.1)	55 (51–59)	1501 (84.3)	0.066
LV ED volume, mL	140 (119–163)	1533 (86.1)	130 (109–155)	1501 (84.3)	< 0.001
LV ES volume, mL	61 (51–74)	1533 (86.1)	58 (47–71)	1501 (84.3)	< 0.001
LV stroke volume, mL	78 (65–90)	1533 (86.1)	72 (60–85)	1501 (84.3)	< 0.001
Cardiac output, L/min (corrected units)	4.7 (4.1–5.5)	1533 (86.1)	4.7 (4.0–5.5)	1501 (84.3)	0.53
LV mass, g	92 (76–109)	1518 (85.2)	91 (77–108)	1518 (85.2)	0.87
LV mass index, g/m^2^	47 (41–53)	1518 (85.2)	47 (41–52)	1518 (85.2)	0.48
LV global average wall thickness, mm	5.9 (5.4–6.5)	1516 (85.1)	6.1 (5.6–6.6)	1516 (85.1)	< 0.001

IQR stands for inter-quartile range; ECG, electrocardiogram; CMR, cardiac magnetic resonance; bpm, beats per minute; LV, left ventricular; EF, ejection fraction; ED, end-diastolic; ES, end-systolic. All continuous variables are distributed non-normally and compared using the Mann–Whitney U-test

**Table 3:** Multivariate multiple linear regression models used to quantify the association of type 2 diabetes with selected ECG and CMR-derived biomarkers. Models are adjusted sequentially for different types of confounding factors. Socio-demographic factors include age, sex, ethnicity; lifestyle factors include body mass index (BMI), smoking; clinical factors include diastolic blood pressure, total cholesterol, triglycerides, C-reactive protein, anti-hypertensive medication and insulin.

Outcome	Model 0 Unadjusted N = 2577		Model 1 Adjusted for socio-demographic factors N = 2577		Model 2 Additionally adjusted for lifestyle factors N = 2544		Model 3 Additionally adjusted for clinical factors N = 2201	
	Coefficient (95% CI)	*p*-value	Coefficient (95% CI)	*p*-value	Coefficient (95% CI)	*p*-value	Coefficient (95% CI)	*p*-value
*ECG*								
Ventricular rate, bpm	4.14 (3.30–4.98)	< 0.001	4.15 (3.31–4.98)	< 0.001	3.84 (3.01–4.67)	< 0.001	3.11 (2.11–4.1)	< 0.001
QRS duration, ms	− 0.76 (− 1.81–0.28)	0.151	− 0.89 (− 1.89–0.10)	0.079	− 1.14 (− 2.14–(− 0.14))	0.026	− 1.81 (− 3.01–(− 0.61))	0.003
QTc interval, ms	2.57 (0.78–4.37)	0.005	2.48 (0.76–4.20)	0.005	1.83 (0.13–3.53)	0.035	0.86 (− 1.22–2.95)	0.416
T-wave offset, ms	− 10.5 (− 12.9–(− 8.0))	< 0.001	− 10.6 (− 13.0–(− 8.2))	< 0.001	− 10.1 (− 12.5–(− 7.6))	< 0.001	− 8.3 (− 11.2–(− 5.3))	< 0.001
T-wave amplitude (V3), mV	− 0.04 (− 0.06–(− 0.02))	< 0.001	− 0.038 (− 0.06–(− 0.02))	< 0.001	− 0.031 (− 0.048–(− 0.014))	< 0.001	− 0.025 (− 0.047–(− 0.004))	0.021
T-wave amplitude (aVL), mV	− 0.01 (− 0.02–(− 0.01))	< 0.001	− 0.01 (− 0.02–(− 0.01))	< 0.001	− 0.01 (− 0.02–(− 0.01))	< 0.001	− 0.005 (− 0.015–0.004)	0.285
J-point amplitude (V3), mV	0.002 (− 0.003–0.006)	0.437	0.002 (− 0.003–0.006)	0.47	0.003 (− 0.001–0.007)	0.198	0.003 (− 0.002–0.008)	0.218
J-point amplitude (aVL), mV	− 0.004 (− 0.007–(− 0.001))	0.007	− 0.004 (− 0.006–(− 0.001))	0.009	− 0.003 (− 0.006–(− 0.001))	0.019	− 0.001 (− 0.004–0.002)	0.573
Sokolow-Lyon, mm	− 0.83 (− 1.34–(− 0.32))	0.001	− 0.80 (− 1.29–(− 0.30))	0.002	− 0.61 (− 1.09–(− 0.12))	0.015	− 0.39 (− 0.99–0.21)	0.199
*CMR*								
LVEF, %	− 0.415 (− 0.952–0.123)	0.131	− 0.371 (− 0.902–0.161)	0.171	− 0.289 (− 0.825–0.247)	0.29	− 0.195 (− 0.839–0.449)	0.553
LV ED volume, mL	− 8.63 (− 15.2–(− 2.09))	0.010	− 8.44 (− 14.8–(− 2.03))	0.010	− 9.11 (− 15.6–(− 2.61))	0.006	− 6.86 (− 15.3–1.55)	0.11
LV ES volume, mL	− 3.47 (− 9.09–2.15)	0.226	− 3.48 (− 9.06–2.1)	0.221	− 3.95 (− 9.62–1.71)	0.171	− 2.79 (− 10.2–4.59)	0.459
LV stroke volume, mL	− 5.18 (− 6.87–(− 3.5))	< 0.001	− 4.98 (− 6.53–(− 3.42))	< 0.001	− 5.17 (− 6.74–(− 3.6))	< 0.001	− 4.11 (− 6.03–(− 2.19))	< 0.001
Cardiac output, L/min	− 0.0517 (− 0.158–0.055)	0.341	− 0.038 (− 0.137–0.061)	0.453	− 0.0622 (− 0.161–0.037)	0.219	− 0.0429 (− 0.165–0.080)	0.493
LV mass, g	0.359 (− 1.38–2.1)	0.687	0.466 (− 0.846–1.78)	0.487	− 0.388 (− 1.59–0.811)	0.525	− 0.165 (− 1.63–1.3)	0.825
LV mass index, g/m^2^	− 0.17 (− 0.83–0.49)	0.612	− 0.16 (− 0.70–0.38)	0.562	− 0.24 (− 0.78–0.31)	0.393	− 0.10 (− 0.77–0.56)	0.763
LV global average wall thickness, mm	0.173 (0.113–0.232)	< 0.001	0.174 (0.125–0.223)	< 0.001	0.139 (0.094–0.183)	< 0.001	0.133 (0.081–0.186)	< 0.001

Bpm stands for beats per minute; LV, left ventricular; EF, ejection fraction; ED, end-diastolic; ES, end-systolic

### Type 2 diabetes is independently associated with reduced stroke volume and increased left ventricular wall thickness

Left ventricular wall thickness was higher in the T2DM cohort (+ 2 mm, 6.1 [5.6–6.6] vs. 5.9 [5.4–6.5] mm) (Table 2), and its association with T2DM was statistically significant in all models (Table 3). Left ventricular stroke volume was lower in the T2DM cohort (− 6 ml, 72 [60–85] vs. 78 [65–90] ml), as were end-diastolic and end-systolic volumes (Table 2). However, the association of T2DM was significant in all models only with stroke volume (Table 3). No statistically significant differences or associations were observed for LVEF nor cardiac output.

### QRS duration has a positive association with cardiovascular outcomes in type 2 diabetes

T2DM individuals who went on to develop CVD (n = 98, Fig. 2) compared to those who did not (n = 1683) were more likely to be male (70% vs. 64%) and more likely to take metformin (57% vs. 53%). In this cohort of subjects with diabetes, there were no changes observed in blood pressure, cholesterol, HbA1c, or eGFR between individuals who do and do not develop CVD. In the T2DM cohort, cases who developed CVD during follow-up (n = 98) had a longer QRS duration (88 [82–98] vs. 86 [80–94] ms, *p* = 0.03) (Supplementary Table 2). There was a statistically significant association between QRS duration and CVD development in all models but the fully adjusted one (Supplementary Table 3). In subjects without T2DM, cases of CVD (n = 98) had a higher Sokolow-Lyon index (23.4 [17.7–27.1] vs. 20.1 [16.2–24.7] mm, *p* = 0.005). We found a statistically significant association between the Sokolow-Lyon index and CVD development in all models (Supplementary Table 3).

### Left ventricular mass, left ventricular mass index and wall thickness have a positive association with cardiovascular outcomes in cohorts with and without type 2 diabetes

In both cohorts, cases with CVD had a higher left ventricular mass (T2DM: 100 [86–107] vs. 91 [76–108] g, *p* = 0.006; no T2DM: 101 [83–118] vs. 92 [76–108] g, *p* < 0.001), a higher LVMI (T2DM: 48.7 [45.3–54.0] vs. 46.4 [41.2–52.1] g/m^2^, *p* = 0.001; no T2DM: 50.3 [43.0–57.5] vs. 46.7 [41.1–52.8] g/m^2^, *p* = 0.001), and a thicker left ventricular wall (T2DM: 6.3 [5.9–6.8] vs. 6.1 [5.6–6.6] mm, *p* = 0.006; no T2DM: 6.3 [5.6–6.9] vs. 5.9 [5.4–6.4] mm, *p* < 0.001) (Supplementary Table 2). All three variables showed a statistically significant association with CVD in all models (Supplementary Table 3).

### Subgroup analyses

#### Sex, age, and body mass index

Similar trends were observed between biomarkers of subjects with and without T2DM, in females (n = 648) and males (n = 1133). Compared to the control cohort, both females and males in the T2DM group exhibited increased ventricular rates, a prolonged QTc interval, an earlier T wave offset, lower T wave amplitudes in leads V3 and aVL, a lower Sokolow-Lyon index, lower end-diastolic, end-systolic, and stroke volumes, and a thicker left ventricular wall (Supplementary Table 5). As expected, baseline biomarker values were different in males and females. Sex-specific results stratified by age and BMI are available in Supplementary Figs. 3 and 4. No notable differences were observed across age groups. However, it is important to note that due to the design of the UK Biobank, the study only included middle-aged and elderly participants. Potential implications of this demographic trait are considered in the Discussion, notably regarding women and the effects of menopause on CVD risk. In different BMI groups, there was a gradual decrease in Sokolow-Lyon index with increasing BMI in both cohorts, likely explained by increased electrical impedance, and a steady increase in wall thickness with increasing BMI (Supplementary Fig. 3e, 3h).

#### Ethnicity

A higher proportion of participants from non-white ethnic backgrounds had T2DM (141 of 189, 74.6%), compared to participants of white ethnicity with T2DM (1640 of 3373, 48.6%). In both ethnicity subgroups, participants with T2DM showed a significant increase in ventricular rate and an earlier T wave offset (Supplementary Table 6). A significant QRS shortening, QTc prolongation, T wave amplitude decrease, Sokolow-Lyon score decrease, lower end-diastolic, end-systolic, and stroke volumes, and lower left ventricular wall thickness were only observed in white participants. In contrast, no significant changes were observed in the non-white subgroup for these biomarkers.

#### Association of HbA1c with ECG and CMR-derived biomarkers

In individuals with T2DM, we found a statistically significant association of higher levels of HbA1c with both a higher ventricular rate and an earlier T wave offset in all models (Supplementary Table 4). As noted earlier, heart rate and T wave offset are strongly correlated, so this consistent association is expected. Higher levels of HbA1c were also associated with a lower T wave amplitude in lead V3 and a lower stroke volume in all but the fully adjusted model. This suggests that, in addition to T2DM status, changes in the glycaemic spectrum also have a significant positive association with variations in ventricular rate and T wave.

## Discussion

This study is the first to provide a large-scale, concurrent analysis of ECG and CMR-derived biomarkers in a population with T2DM and no prior history of CVD, compared to matched controls. Individuals in the T2DM cohort had a higher resting ventricular rate, prolonged QTc interval, reduced T wave amplitude, thicker left ventricular wall and lower end-diastolic, end-systolic and stroke volumes. T2DM was associated with all changes, notably showing a strong independent association with heart rate, left ventricular wall thickness and stroke volume after full model adjustment. These findings may reflect subclinical diabetes-induced pathophysiological changes, notably the presence of diabetic cardiomyopathy, which has been debated for many years [2, 5]. We discuss the clinical implications of these findings below.

### Implications of a higher resting heart rate and QTc prolongation on cardiac autonomic neuropathy and arrhythmias

T2DM showed a strong, independent association with a higher resting heart rate, even after adjustment for potential confounders including medication. High heart rates have been associated to hypertension and metabolic conditions for many decades [28, 29]. In T2DM subjects specifically, this is typically a consequence of cardiac autonomic neuropathy [9]. Cardiac autonomic neuropathy causes heart rate to increase in the early stages of disease and gradually decrease back to normal, but with reduced variability and an increased likelihood of arrhythmias [30]. This may explain why heart rates in our T2DM cohort lie mostly within the normal clinical range of 60–100 beats per minute. Additionally, we found a significant association between HbA1c and resting heart rate (Supplementary Table 4). This supports evidence linking dysregulations in glucose metabolism with increased sympathetic nervous system activity and severity of cardiac autonomic neuropathy [31–33].

T2DM was also associated with a longer QTc interval. The strength of this association decreased after adjusting for clinical covariates, suggesting that other factors may be partly responsible for the observed QTc interval prolongation. In the T2DM cohort, 113 women and 159 men (n = 272 of 1781 [15%]) met sex- and age-specific criteria for clinical QTc prolongation [34], compared to n = 211 (12%) in the control cohort. QTc prolongation is a well-established risk factor for cardiac arrhythmias and is correlated with a higher cardiac autonomic neuropathy score in patients with T2DM [14]. Diabetic cardiomyocytes have a local renin-angiotensin system; when activated, increases in angiotensin II causes a reduction of transient outward potassium current Ito, extending the action potential repolarisation phase and thus prolonging the QT interval [7, 35]. In addition, high glucose decreases hERG channel expression, which in turn modulates the delayed rectifier potassium current IKr, another factor responsible for QT prolongation in diabetes [36, 37].

### T wave amplitude reduction, asymptomatic ischemic heart disease and metabolic disturbances

T2DM subjects exhibited lower T wave amplitudes in leads V3 and aVL. BMI was included both as a matching variable and as a model covariate, to minimise the effect of body composition on electrical impedance and subsequent reductions in ECG amplitude. Thus, our results suggest a plausible direct effect of T2DM on T wave amplitude. A recent ECG study also showed that T wave amplitudes were reduced in individuals with T2DM [38]. However, some subjects in this study had a history of myocardial infarction, which itself is likely to affect the T wave amplitude; our study bypasses this limitation as our study sample is free of past diagnosed cardiovascular conditions. That being said, asymptomatic ischemic heart disease may be partly responsible for changes observed. A reduced T wave amplitude has previously been associated with hypokalaemia, as well as hyperinsulinemic hypoglycaemia, which is common in T2DM [39, 40]. Thus, the lower T wave amplitudes observed may reflect underlying changes in potassium, insulin or glucose levels, or silent ischemia.

### Lower stroke volume and thicker ventricular wall, subtle changes in the trajectory of heart failure with preserved ejection fraction

We observed a significant decrease in stroke volume in the T2DM cohort, accompanying the increase in heart rate, while no change was observed in cardiac output. A previous study examined hemodynamic parameters, including stroke volume, in a smaller cohort of 143 subjects with type 1 and 2 diabetes [10]. Our results, based on a much larger matched cohort with type 2-specific diabetes, corroborate these findings. We also found that T2DM was associated with a thicker left ventricular wall, independently of blood pressure. This is consistent with previous studies that have demonstrated that patients with diabetes, hypertension, or both, tend to develop a thicker myocardial wall [41–43]. In later stages of disease, left ventricular hypertrophy is common in T2DM patients due to increased myocardial steatosis leading to hypertrophic signalling and concentric remodelling [11]. Patients with heart failure with preserved ejection fraction (HFpEF), a frequent and serious complication of T2DM, also tend to have a lower stroke volume and increased left ventricular wall thickness [44, 45]. The changes observed in our study may represent early, subclinical stages of adverse remodelling; measurements of left ventricular end-diastolic volume in our T2DM cohort remained within the normal sex-specific reference ranges that have been established for healthy adults without diabetes in the UK Biobank [23]. In the T2DM cohort, median wall thickness (6.1 mm) remained well below the clinical threshold for left ventricular hypertrophy (end-diastolic maximal wall thickness ≥ 15.0 mm) [46], and the absolute change compared to the control cohort was subtle (+ 0.2 mm). Despite a thicker left ventricular wall, the Sokolow-Lyon index was lower in the T2DM cohort, suggesting lower R and S wave amplitudes. The discordance between this index and left ventricular hypertrophy as assessed by CMR is interesting but plausible. In obese patients, QRS voltages are artificially reduced due to increased body fat causing electrical impedance [18]. Despite having similar BMI and left ventricular mass, subjects in different cohorts may have varying chest wall shapes, or additional myocardial fat deposition contributing to non-electrically active left ventricular mass, which may drive the observed difference in Sokolow-Lyon index.

### Increased QRS duration, higher left ventricular mass and higher left ventricular wall thickness are associated with the development of cardiovascular disease in type 2 diabetes

Considering CVD outcomes in the T2DM cohort, no statistically significant differences were observed in blood pressure, cholesterol, HbA1c, or eGFR between subjects who did and did not develop CVD. These biomarkers are clinical risk factors used in the calculation of SCORE2-Diabetes, a 10-year T2DM-specific cardiovascular risk prediction score developed by a working group of the European Society of Cardiology [47]. Here, the lack of significant differences may be due to a shorter follow-up time or a small sample size (n = 98 CVD cases). Changes in ECG biomarkers between CVD cases and controls differed in the cohorts with and without T2DM (Supplementary Table 2). In the T2DM cohort, 668 subjects (38%) had a heart rate above 70 bpm, a threshold associated with a higher risk of cardiovascular events specifically in T2DM [48], however we did not observe significant changes in heart rate between cases and controls in both cohorts. An increased QRS duration was the only ECG biomarker significantly associated with the development of CVD in the T2DM cohort. In the ACCORD trial, QRS duration was increased in patients with diabetes and incident heart failure (HF) compared to those without HF [49]. Additionally, a longer QRS complex was an independent predictor of cardiovascular events in middle- to older-aged men and is associated with all-cause mortality in T2DM [50, 51]. In contrast, in the control cohort, the Sokolow-Lyon index was the only ECG biomarker showing a significant association with the development of future CVD. This is in line with previous studies linking left ventricular hypertrophy with adverse cardiovascular events [52–56]. The hypothesis behind the hypertrophic CVD phenotype is strengthened by the strong independent association of increased left ventricular mass, LVMI and wall thickness with development of CVD, which was present in both cohorts.

### Sex, age, body mass index and ethnicity-specific differences

In male and female subgroups, baseline biomarker values differed, which is expected [57–59]. The sex-specific subgroup analysis suggests that trends according to T2DM status were consistent across both sexes. Little to no differences in biomarkers were observed among different age groups, however this result is unlikely to generalise to younger individuals outside the age range of this study (< 40 years old), particularly women. Evidence suggests that post-menopausal women, and those in the menopausal transition phase, are at higher risk of CVD compared to those in their reproductive years [60]. This risk is further increased by an earlier age at menopause, Black ethnicity, and T2DM [61]. Fluctuations in hormones and lipid profile that occur during menopause may affect cardiac function and electrophysiology, however this was a challenge to assess in this study, given that women recruited in the UK Biobank were all above 40 years old, i.e. already in the peri- or post-menopause stages. Moreover, we found a notable imbalance of ethnicity groups, with 1733 (97.3%) and 1640 (92.1%) of all participants with and without T2DM, respectively, being from a white ethnic background (Table 1). Although this is reasonably representative of the national distribution for different ethnic groups in the UK population at the time of recruitment [62], the small sample size of individuals from a non-white ethnic background (n = 189) may have driven the lack of significant differences found between those individuals with (n = 141) and without T2DM (n = 48) (Supplementary Table 6). A larger, more ethnically diverse cohort spanning a wider age range would be beneficial to investigate age- and ethnicity-related differences, especially in women.

### Strengths and limitations

The size of the study sample used strengthens the credibility of our results. Thanks to the range of data available in the UK Biobank, we were able to characterise our cohorts in depth by capturing detailed demographical and clinical data of all participants studied. These data were used to match the exposure and control groups, and were included as covariates in our regression analyses, thus accounting for any confounding effects on biomarker outcomes. The UK Biobank’s robustly validated and systematic data recording protocol ensured that data collection bias was minimised [62].

Our study also has several limitations. Firstly, statistical significance does not necessarily equate to clinical relevance. Slight changes in certain biomarkers may not be much larger than natural population variations. This is particularly relevant to subgroup analyses involving smaller sample sizes, which may result in imprecise estimates due to random error. This applies specifically to groups with CVD outcomes (n = 98) and non-white ethnicity (n = 289, of which n = 48 without T2DM). Regardless of sample size, we strived to interpret all statistically significant results cautiously and within the context of existing knowledge established by previously published research. The broad UK Biobank medication category used in the study does not contain information on specific compounds, dose, or treatment duration. This limits our ability to assess the impact of common drugs such as beta-blockers on our results, notably ECG biomarkers, which may cause the effect of T2DM to be underestimated. This could be explored in future work, possibly using a dataset with more comprehensive information on medication. Another limitation is the lack of widespread UK Biobank linkage to primary care records. As of 2024, only 45% of the UK Biobank cohort was linked to these records for general research purposes [63]. This directly impacts cohort size and composition, especially for chronic conditions like diabetes which tend to be diagnosed and recorded within a primary care setting as opposed to hospital admissions. Linkage for the entire UK Biobank cohort would increase statistical power and robustness of future population studies. We believe that matching participants by BMI was adequate for previously stated reasons, however we acknowledge that waist-to-hip ratio is a better indicator of general health and mortality [64]. Finally, we recognise that including multiple distinct conditions within the broad umbrella of CVD may dissolve opposing trends in certain markers. This could be tackled by focusing on a single condition, albeit with small sample sizes.

## Conclusion

Our study examined multi-modal cardiac biomarkers of a large cohort of individuals with type 2 diabetes, a well-established high-risk factor for the development of cardiovascular disease. Despite a lack of diagnosis of cardiovascular disease in all subjects at the time of assessment, type 2 diabetes was associated with an increased resting ventricular rate and an altered ECG including a prolonged QTc interval and lower T wave amplitude, compared to controls matched by sex, age and body mass index. ECG changes were accompanied by an independent association of type 2 diabetes with a decrease in stroke volume and thicker ventricular wall. Our results provide strong evidence supporting current hypotheses on diabetes-induced pathophysiological alterations in the heart, notably diabetic cardiomyopathy and the development of a pro-arrhythmic substrate. Subject to further validation, our findings support the importance of using ECG and cardiac imaging to identify subclinical cardiac abnormalities in patients with type 2 diabetes, ultimately aiming to improve cardiovascular risk management in this high-risk population.

## Supplementary Information

Supplementary Figures

Supplementary Tables

## Data Availability

The data underlying this study were provided by the UK Biobank upon application. Access to UK Biobank data for research purposes can be obtained upon application (https://www.ukbiobank.ac.uk/). This study was conducted under UK Biobank Application Number 40161.
