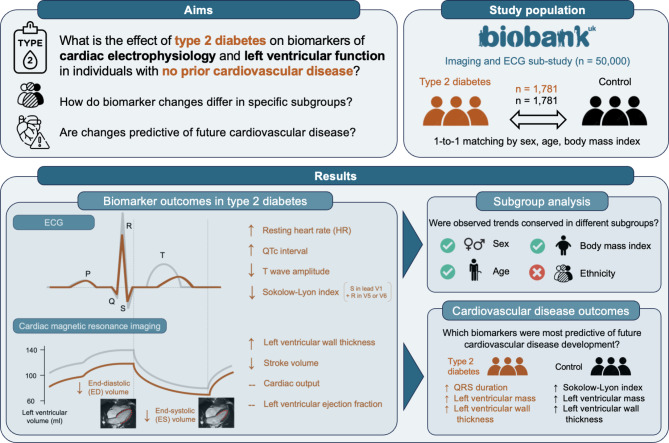# Correction: Multi-modal characterisation of early-stage, subclinical cardiac deterioration in patients with type 2 diabetes

**DOI:** 10.1186/s12933-024-02563-x

**Published:** 2025-01-29

**Authors:** Ambre Bertrand, Andrew Lewis, Julia Camps, Vicente Grau, Blanca Rodriguez

**Affiliations:** 1 Computational Cardiovascular Science Group, Department of Computer Science, University of Oxford, Oxford, OX1 3QD UK; 2 Division of Cardiovascular Medicine, Radcliffe Department of Medicine, University of Oxford, Oxford, OX3 9DU UK; 3 Institute of Biomedical Engineering, Department of Engineering Science, University of Oxford, Oxford, OX3 7DQ UK

**Correction to: Cardiovascular Diabetology (2024) 23:371** 10.1186/s12933-024-02465-y

Following publication of the original article [1], the graphic abstract was missing from this article and should have appeared as shown below. The original article has been updated.

## Graphical Abstract