# Supplementary material for: Multi-modal characterisation of early-stage, subclinical cardiac deterioration in patients with type 2 diabetes
Source: Cardiovasc Diabetol. 2024 Oct 19;23:371. doi: 10.1186/s12933-024-02465-y (PMC11491016; doi:10.1186/s12933-024-02465-y)
Supplement: Supplementary file 1 — Supplementary Figures [file 12933_2024_2465_MOESM1_ESM.docx]

**Figure 1.** Matrix of Pearson correlation coefficients for 12-lead ECG amplitude measurements of T wave amplitude (a) and J point amplitude (b). Coefficients were computed on 2702 (a) and 3562 cases (b), respectively. These cases combine participants from both the type 2 diabetes and control cohorts, and contained no missing data among the features of interest.


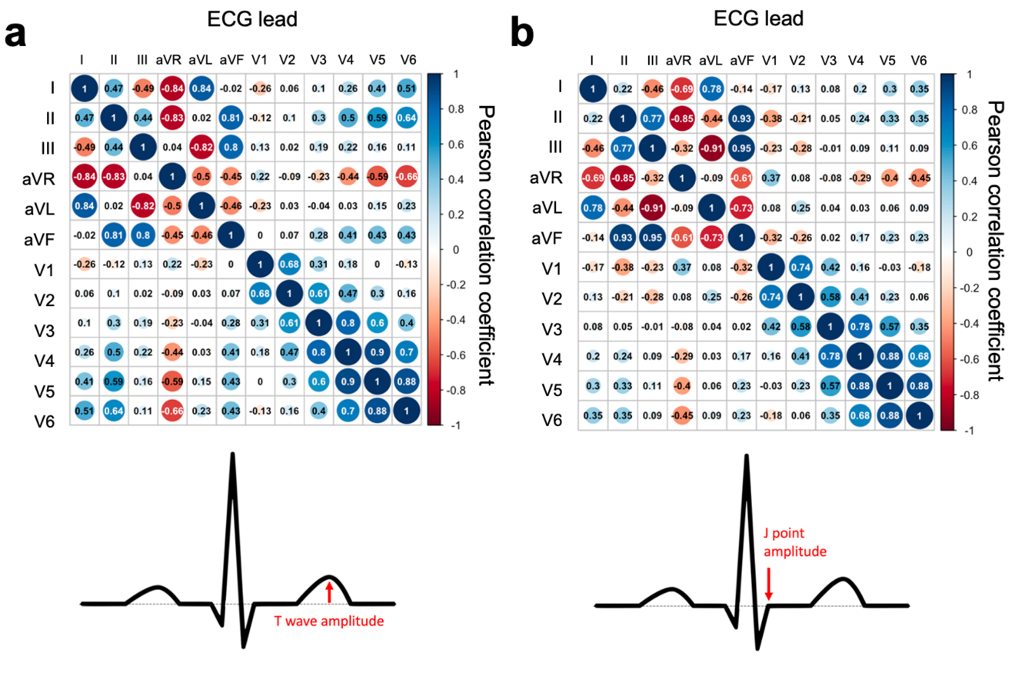


**Figure 2.** Matrix of Pearson correlation coefficients between baseline socio-demographic, lifestyle, and clinical measurements. Coefficients were computed on 2620 cases. These cases combine participants from both the type 2 diabetes and control cohorts, and contained no missing data among the features of interest. BMI: body mass index, HDL: high density lipoprotein, eGFR: estimated glomerular filtration rate.


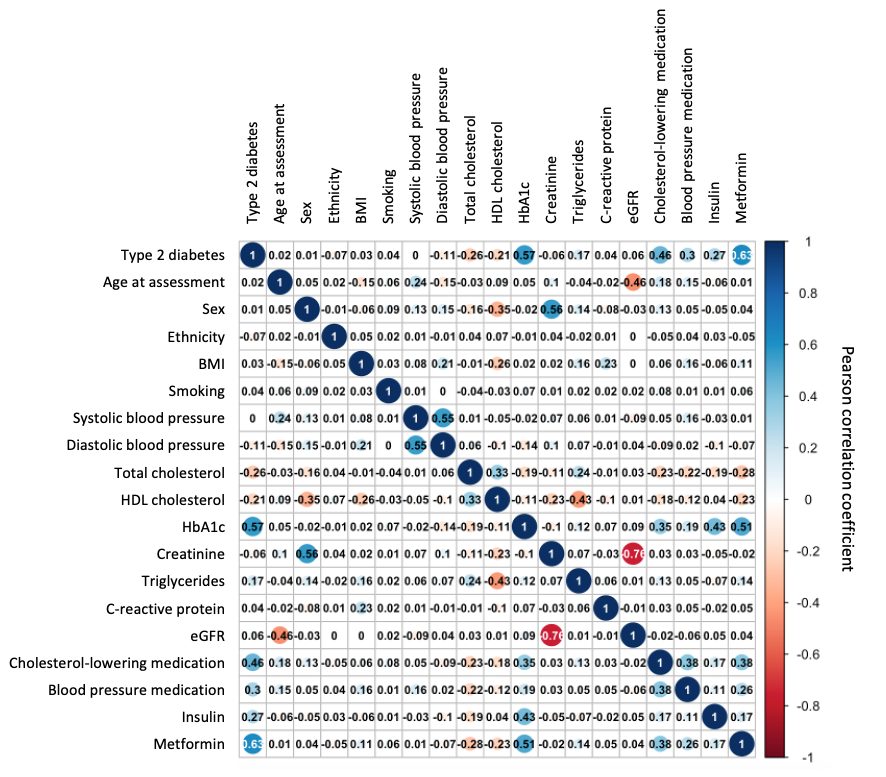


**Figure 3.** Distribution of sex-specific ECG and CMR-derived biomarkers stratified by age. Bpm: beats per minute, LV: left ventricular, EF: ejection fraction. Cases with LVEF <20%, QTc interval >600ms and LV stroke volume >300ml are considered outliers and are not shown in the plots.


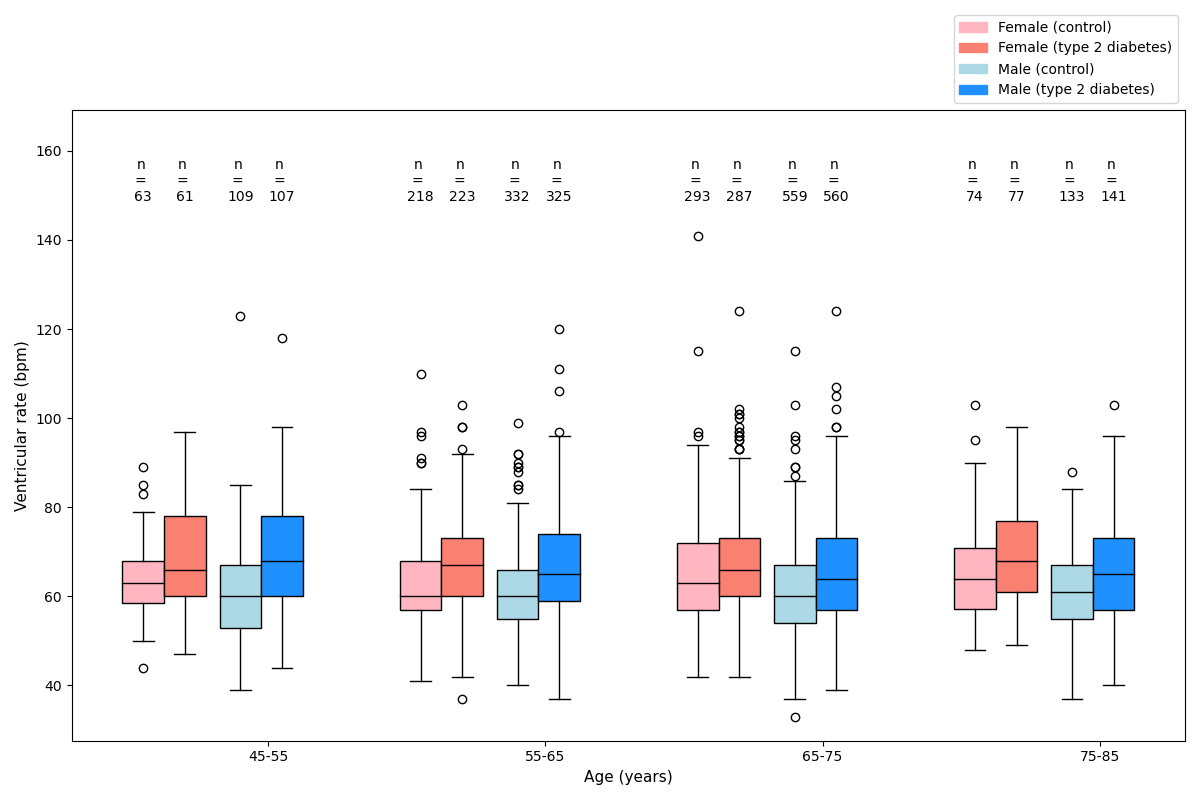


**a**


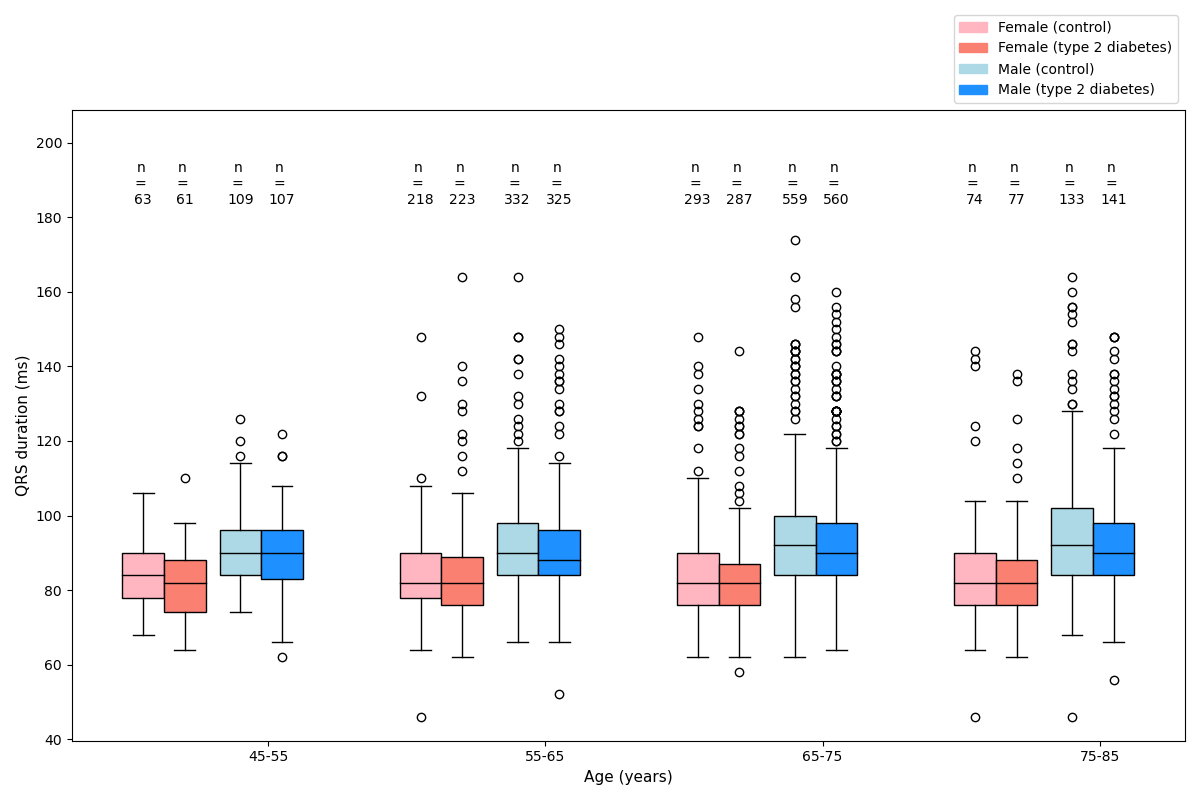


**b**


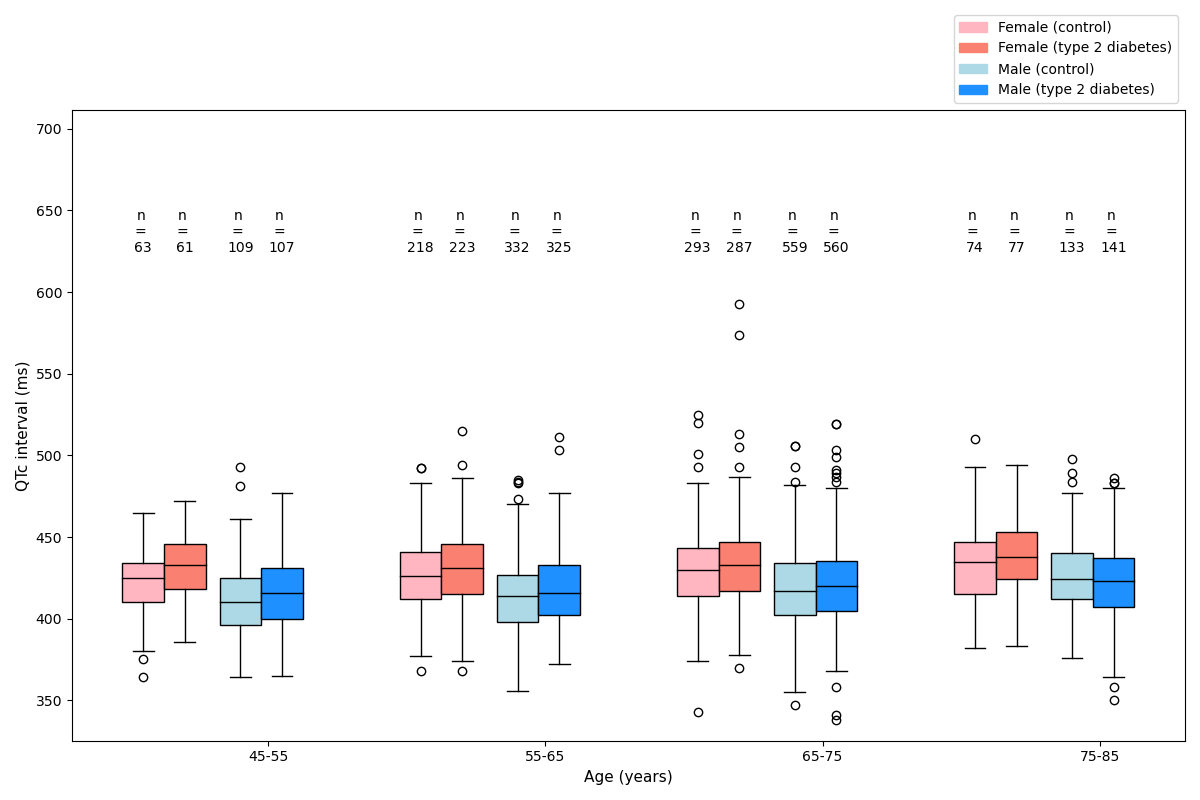


**c**


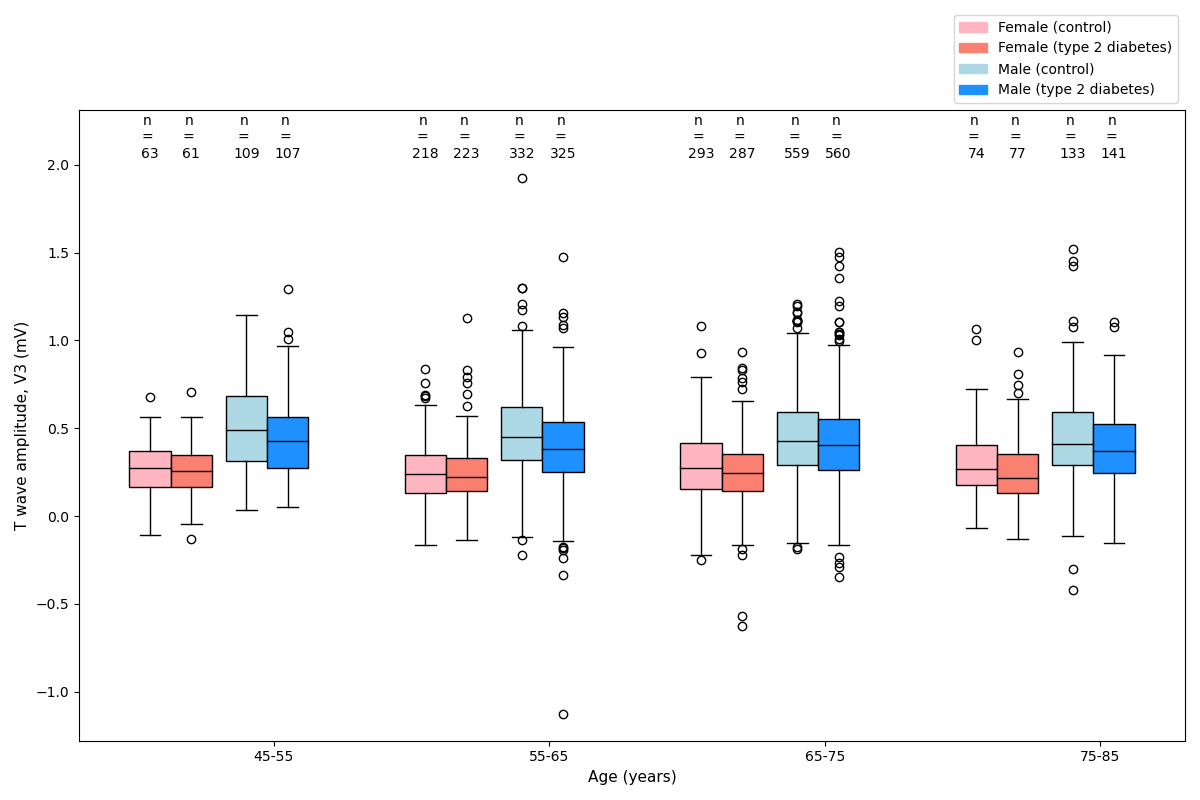


**d**


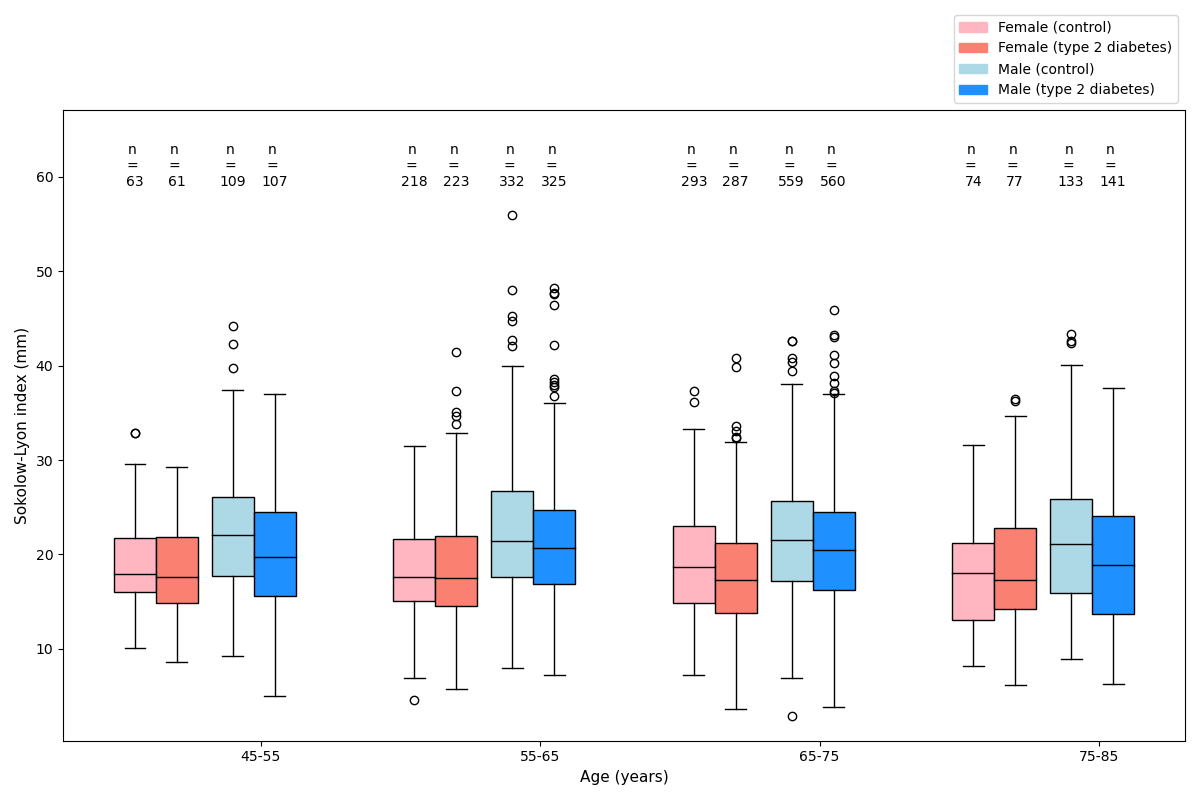


**e**


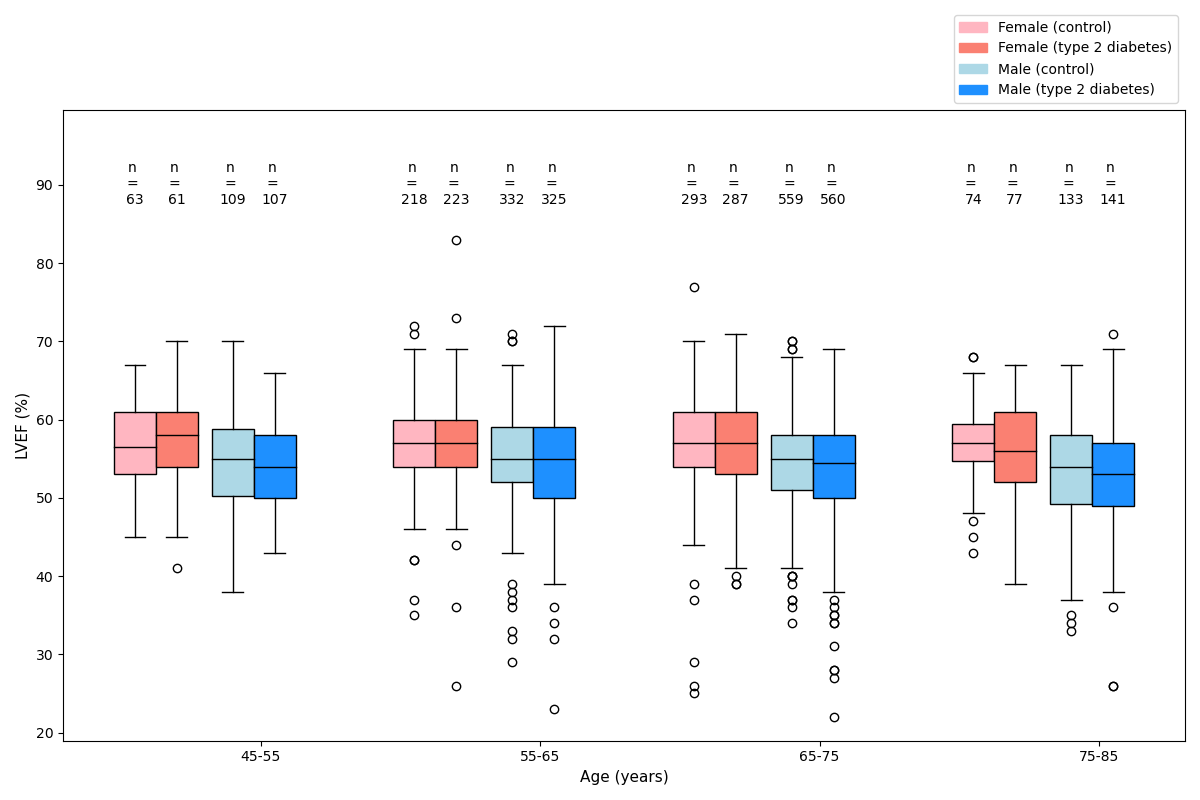


**f**


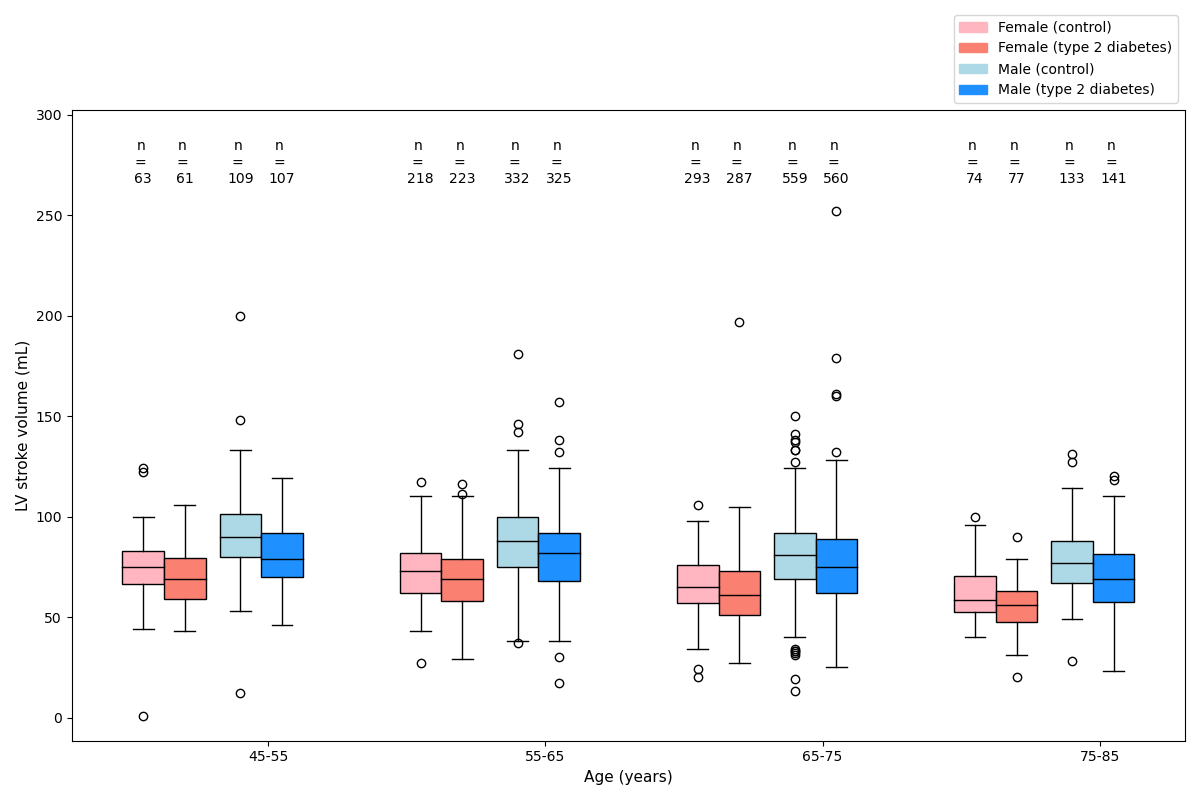


**g**


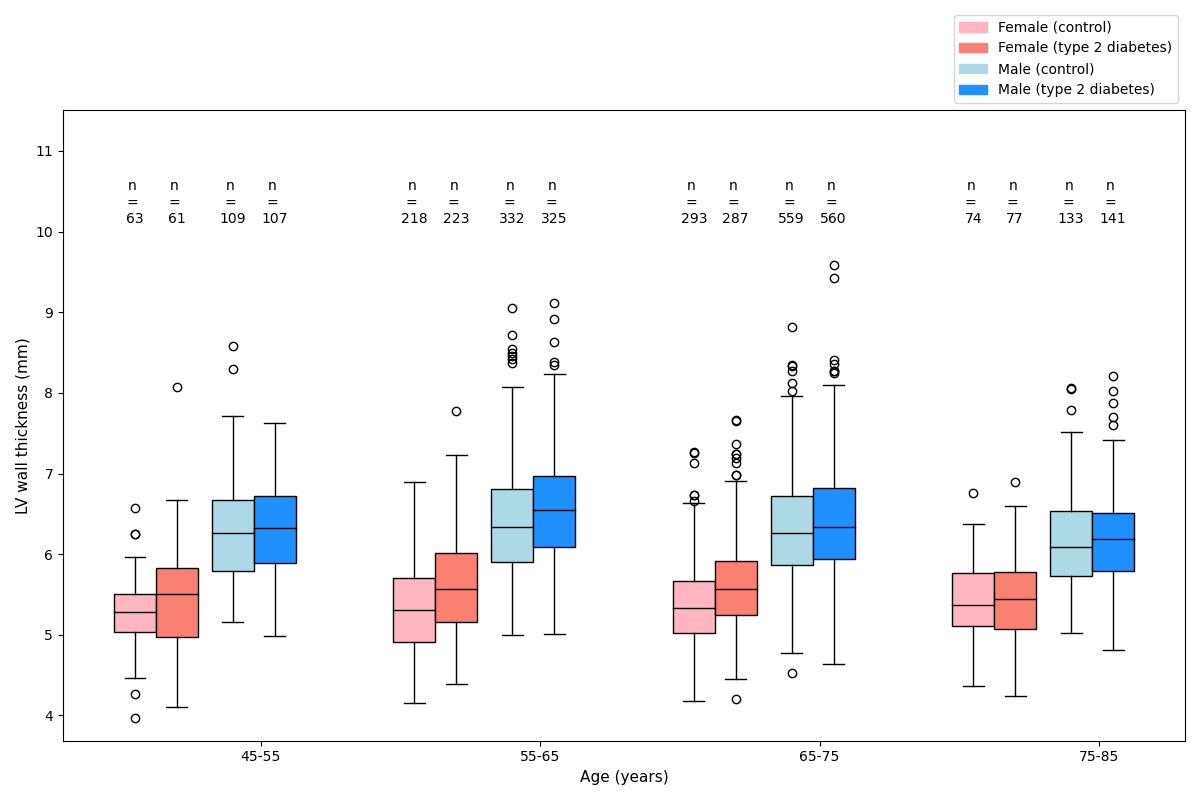


**h**

**Figure 4.** Distribution of sex-specific ECG and CMR-derived biomarkers stratified by body mass index. Bpm: beats per minute, LV: left ventricular, EF: ejection fraction. Cases with LVEF <20%, QTc interval >600ms and LV stroke volume >300ml are considered outliers and are not shown in the plots.


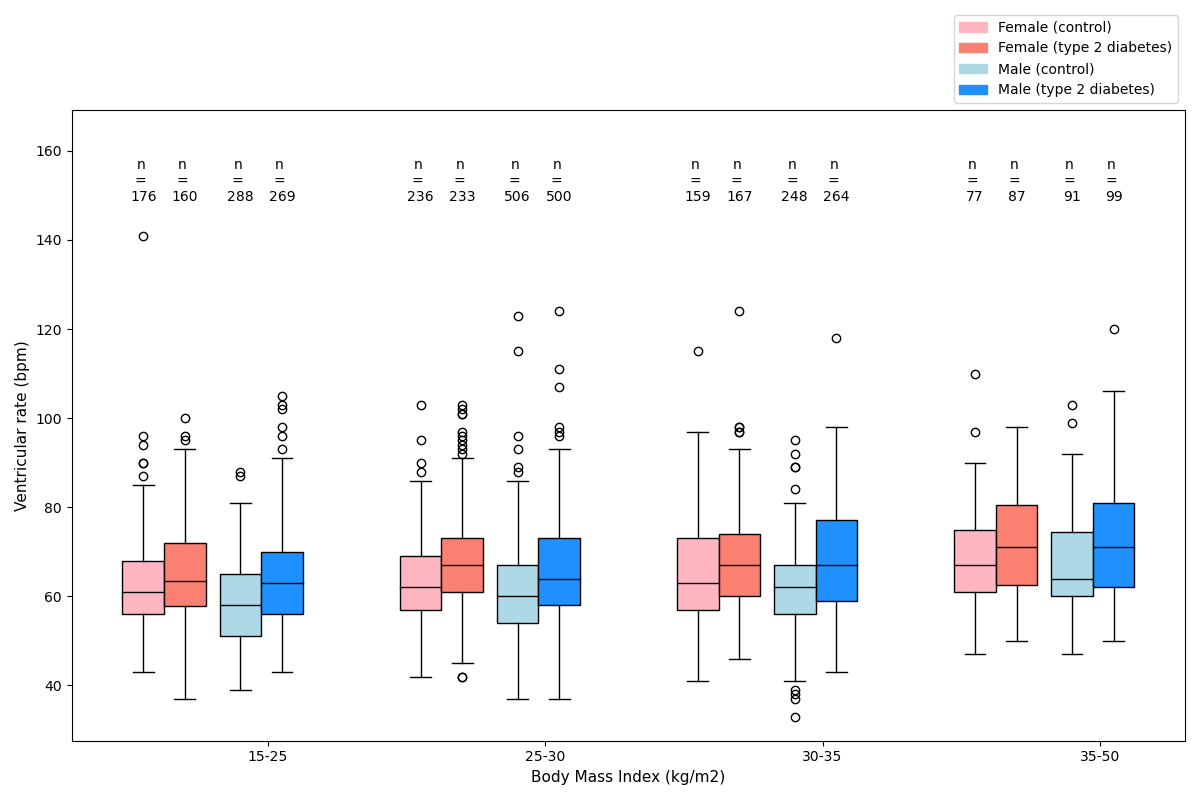


**a**


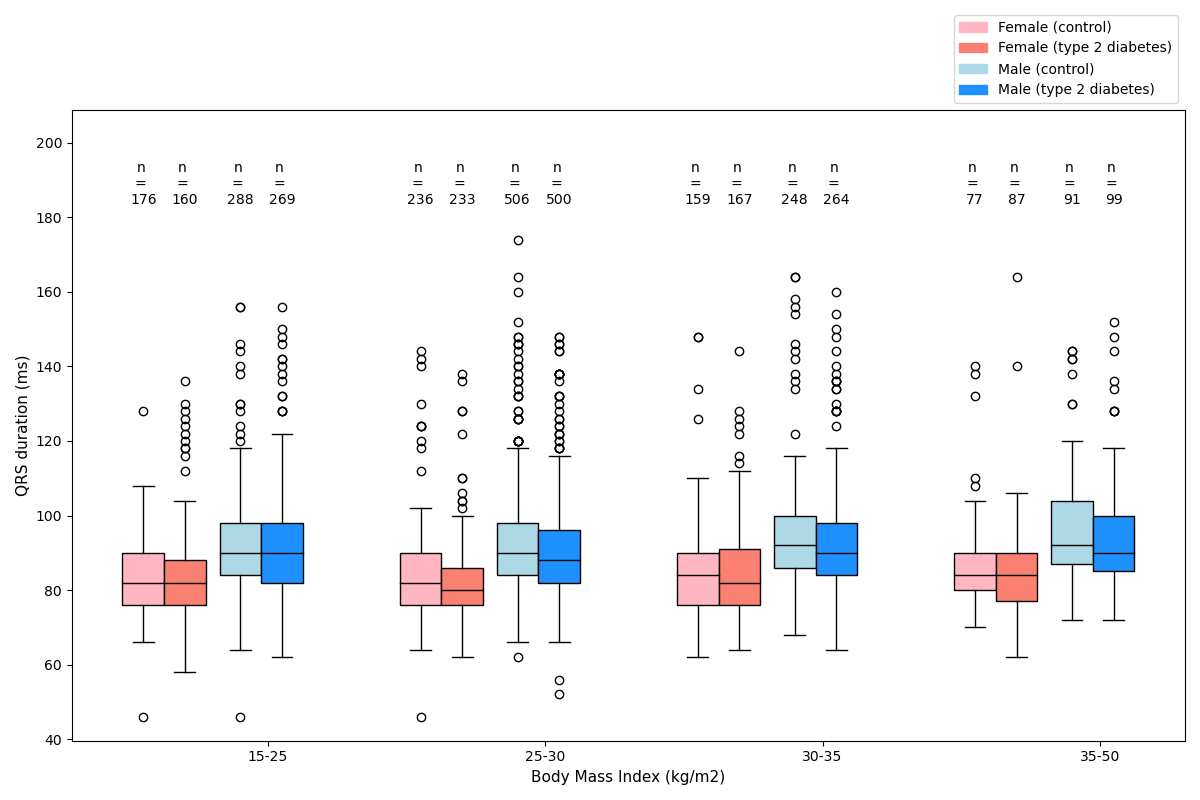


**b**


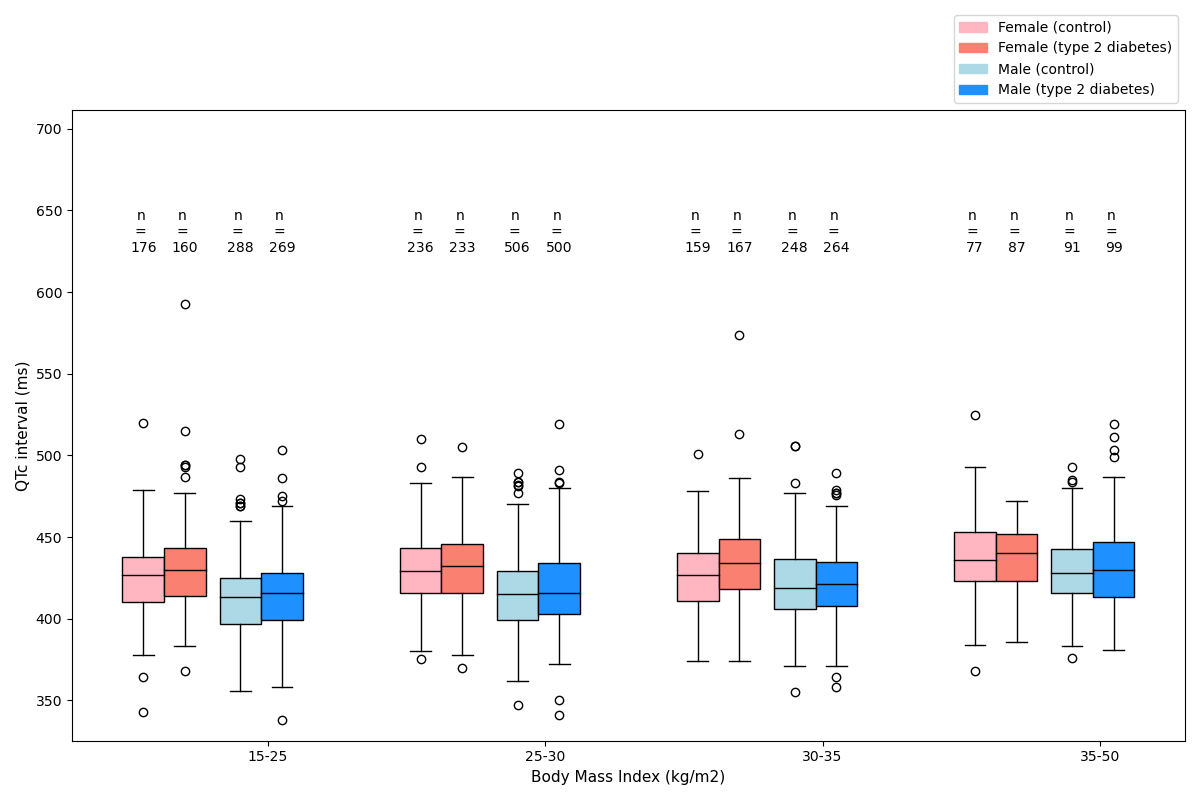


**c**


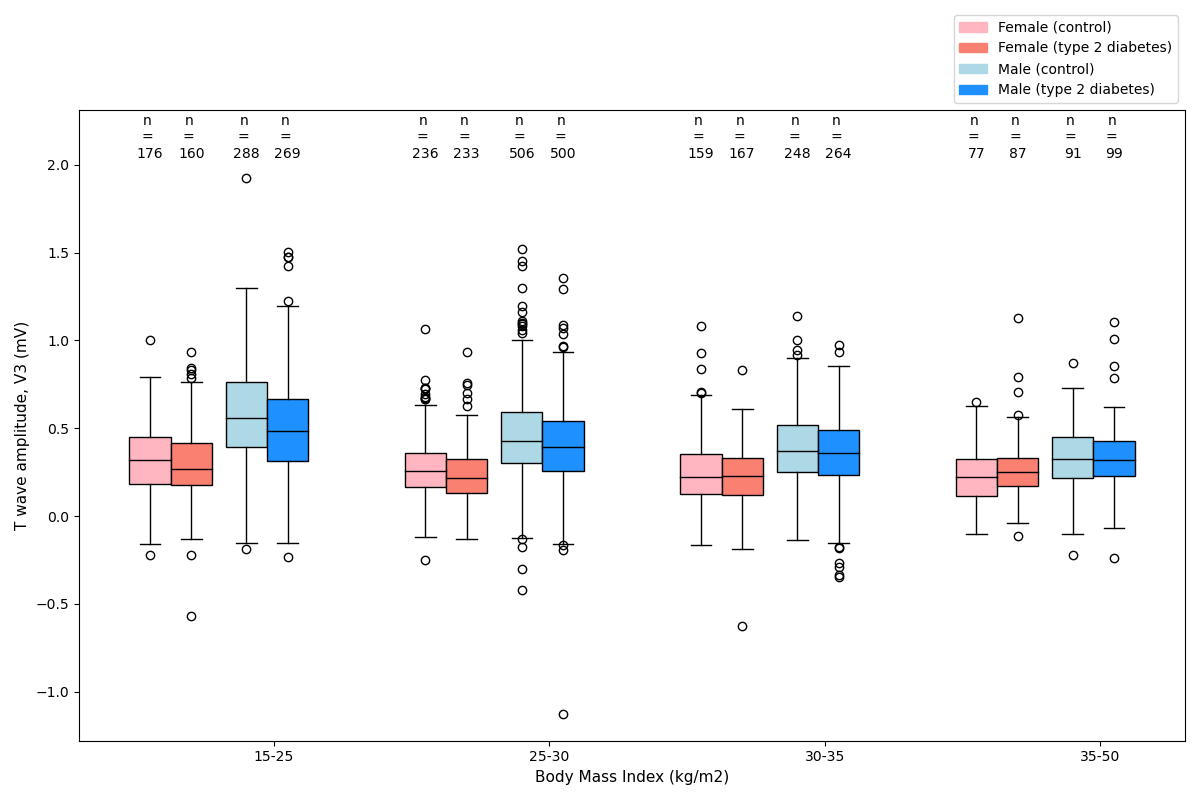


**d**


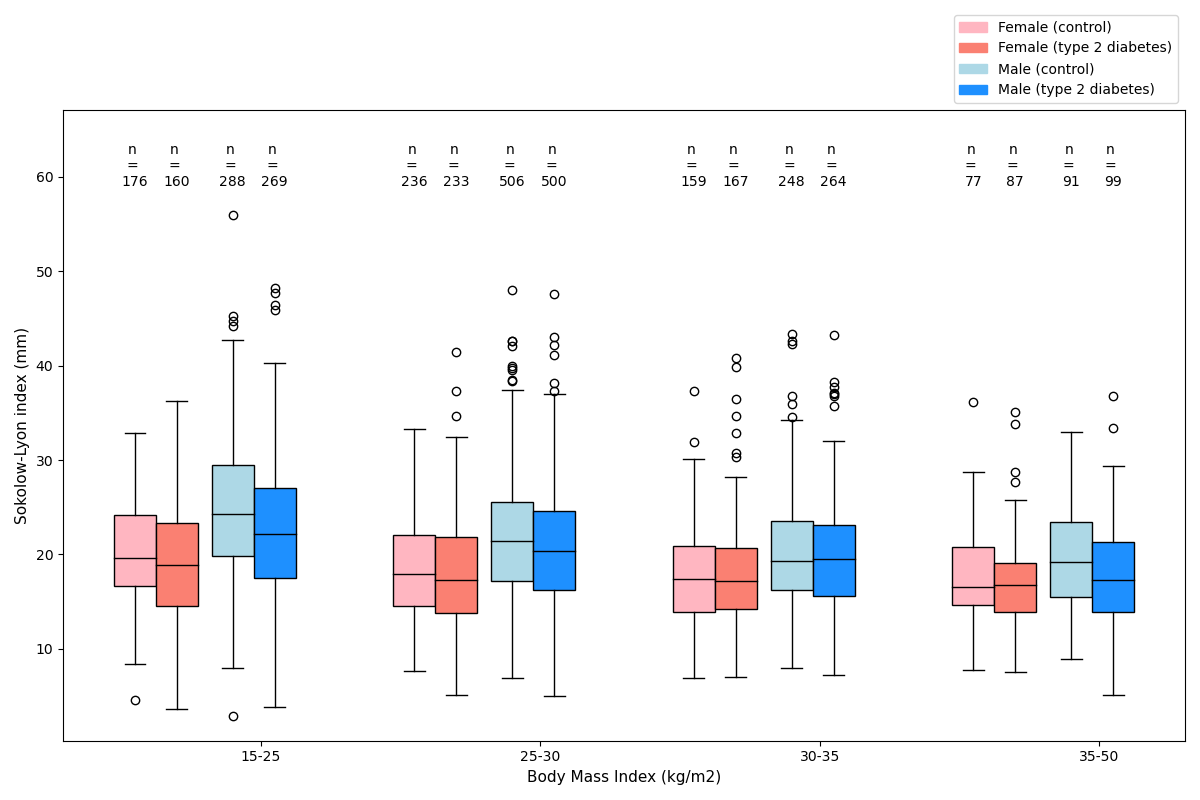


**e**


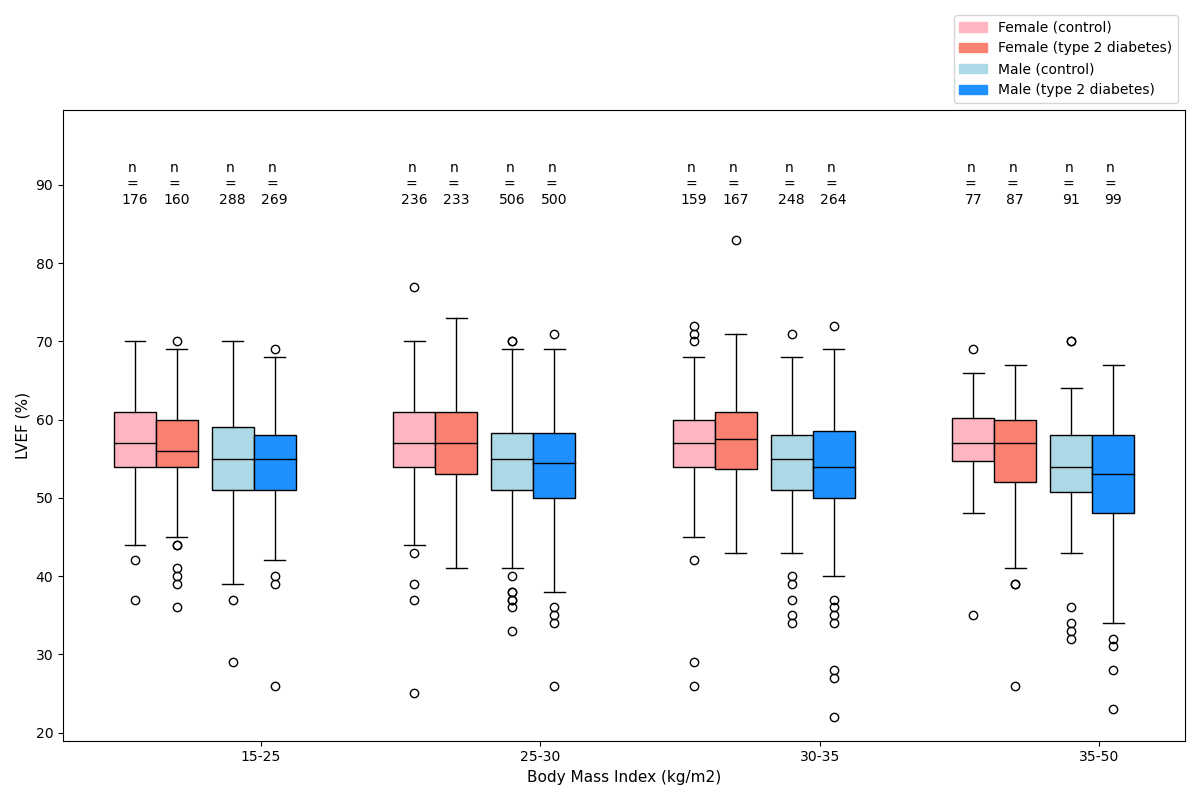


**f**


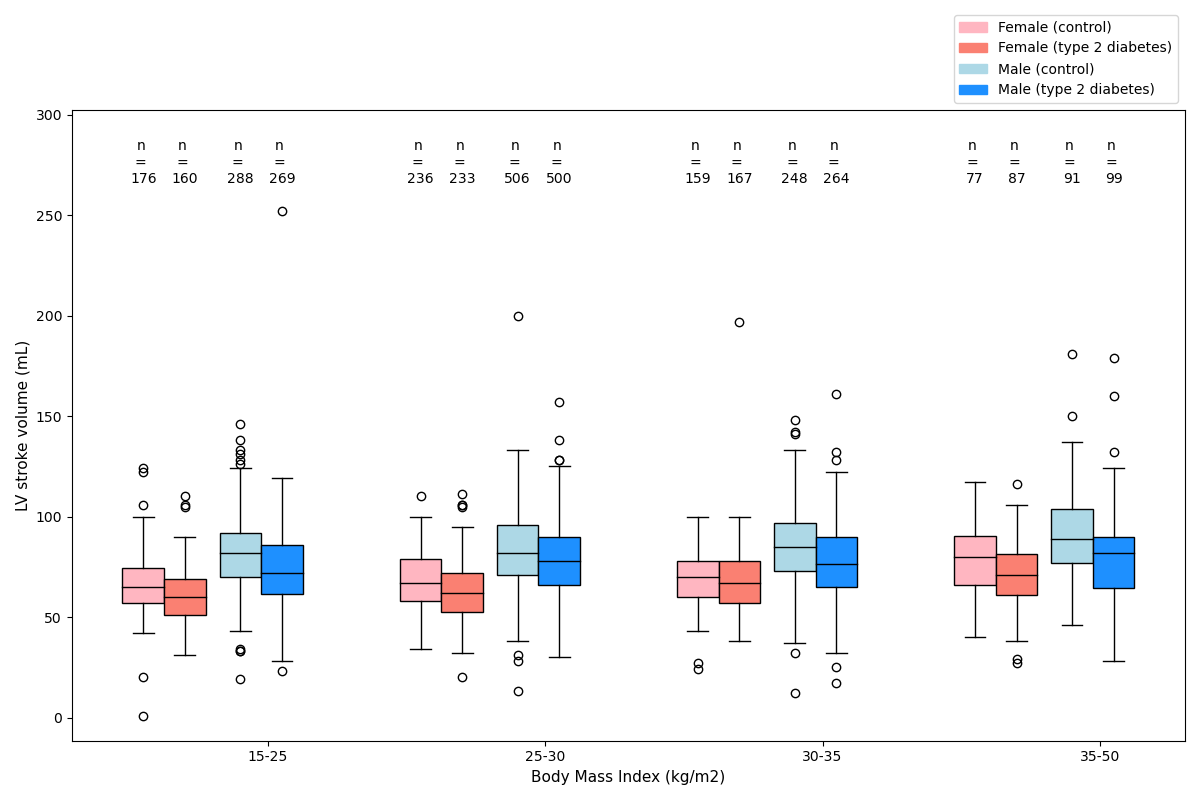

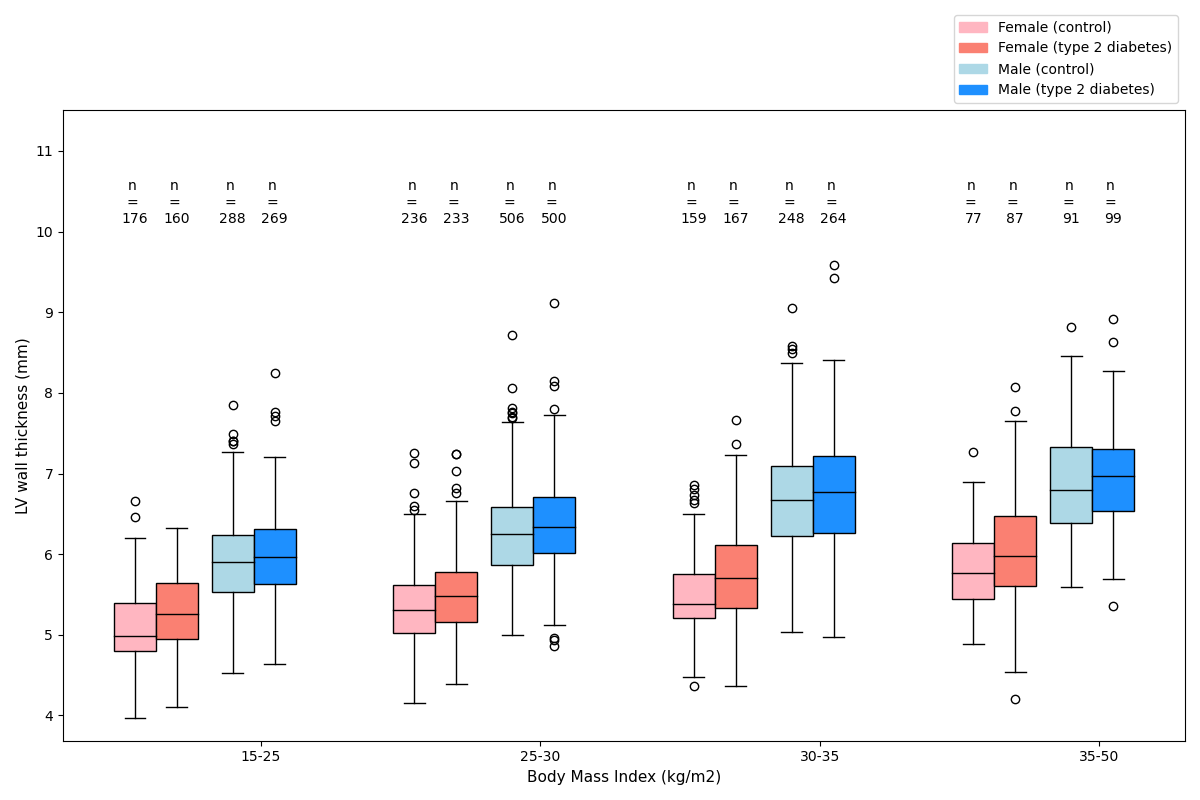


**h**

**g**
