# Supplementary material for: Multi-modal characterisation of early-stage, subclinical cardiac deterioration in patients with type 2 diabetes
Source: Cardiovasc Diabetol. 2024 Oct 19;23:371. doi: 10.1186/s12933-024-02465-y (PMC11491016; doi:10.1186/s12933-024-02465-y)
Supplement: Supplementary file 2 — Supplementary Tables [file 12933_2024_2465_MOESM2_ESM.docx]

**Table 1.** Selected ICD-9 and ICD-10 codes for type 2 diabetes and cardiovascular disease used to define the study cohorts. UK Biobank fields corresponding to the first reported date of ICD-10 code are indicated in the right column. No equivalent fields exist for ICD-9 codes.

| **ICD-X code** | | **Description** | **UK Biobank data field for**  **“Date [ICD-10 code] first reported”** |
| --- | --- | --- | --- |
| **Type 2 diabetes** | | | |
| ICD-9 | 250.x0 | diabetes mellitus, type II or unspecified type, not stated as uncontrolled | - |
|  | 250.x2 | diabetes mellitus, type II or unspecified type, uncontrolled | - |
| ICD-10 | E11 | type 2 diabetes mellitus | 130708 |
|  | E14 | unspecified diabetes mellitus | 130714 |
| **Cardiovascular disease** | | | |
| ICD-9 | 410 | Acute myocardial infarction | - |
|  | 411 | Other acute and subacute forms of ischaemic heart disease | - |
|  | 412 | Old myocardial infarction | - |
|  | 413 | Angina pectoris | - |
|  | 414 | Other forms of chronic ischaemic heart disease | - |
|  | 425 | Cardiomyopathy | - |
|  | 426 | Conduction disorders | - |
|  | 427 | Cardiac dysrhythmias | - |
|  | 428 | Heart failure | - |
| ICD-10 | I20 | Angina pectoris | 131296 |
|  | I21 | Acute myocardial infarction | 131298 |
|  | I22 | Subsequent myocardial infarction | 131300 |
|  | I24 | Other acute ischaemic heart diseases | 131304 |
|  | I25 | Chronic ischaemic heart disease (including I25. 1: Atherosclerotic heart disease of native coronary artery, a.k.a. coronary artery disease (CAD)) | 131306 |
|  | I42 | Cardiomyopathy | 131338 |
|  | I44 | Atrioventricular and left bundle-branch block | 131342 |
|  | I46 | Cardiac arrest | 131346 |
|  | I47 | Paroxysmal tachycardia | 131348 |
|  | I48 | Atrial fibrillation and flutter | 131350 |
|  | I49 | Other cardiac arrhythmias | 131352 |
|  | I50 | Heart failure | 131354 |

**Table 2.** Biomarker differences for participants with and without incident cardiovascular disease. IQR: inter-quartile range, LV: left ventricular, EF: ejection fraction, ED, end diastolic; ES, end systolic. All variables are distributed non-normally and compared using the Mann-Whitney U-test.

|  | **No incident CVD** | | **Incident CVD** | |  |
| --- | --- | --- | --- | --- | --- |
| **Outcome** | **Median (IQR)** | **N (%)** | **Median (IQR)** | **N (%)** | **p-value** |
| **Cohort: no type 2 diabetes** | | | | | |
| Ventricular rate, bpm | 61 (55-68) | 1683 (100) | 62 (55.2-72.2) | 98 (100) | 0.218 |
| QRS duration, ms | 88 (82-96) | 1683 (100) | 90 (82-98) | 98 (100) | 0.078 |
| QTc interval, ms | 420 (404-436) | 1683 (100) | 422 (413-444) | 98 (100) | 0.100 |
| T-wave offset, ms | 854 (834-874) | 1683 (100) | 848 (824-872) | 98 (100) | 0.140 |
| J-point amplitude (V3), mV | -0.015 (-0.044-0.019) | 1683 (100) | 0 (-0.0387-0.034) | 98 (100) | 0.031 |
| T-wave amplitude (V3), mV | 0.366 (0.229-0.533) | 1668 (99.1) | 0.407 (0.257-0.59) | 98 (100) | 0.076 |
| J-point amplitude (aVL), mV | 0.009 (-0.01-0.029) | 1683 (100) | 0.009 (-0.015-0.029) | 98 (100) | 0.570 |
| T-wave amplitude (aVL), mV | 0.112 (0.058-0.166) | 1623 (96.4) | 0.092 (0.019-0.156) | 95 (96.9) | 0.049 |
| Sokolow-Lyon index, mm | 20.1 (16.2-24.6) | 1564 (92.9) | 23.4 (17.7-27.1) | 86 (87.8) | 0.005 |
| LVEF, % | 56 (52-59) | 1445 (85.9) | 56 (50-60.2) | 88 (89.8) | 0.682 |
| LV ED volume, ml | 140 (119-162) | 1445 (85.9) | 142 (120-167) | 88 (89.8) | 0.437 |
| LV ES volume, ml | 61 (51-73) | 1445 (85.9) | 61 (50-78.2) | 88 (89.8) | 0.447 |
| LV stroke volume, ml | 78 (65-90) | 1445 (85.9) | 79 (65.8-88.2) | 88 (89.8) | 0.953 |
| Cardiac output, L/min | 4.7 (4-5.6) | 1445 (85.9) | 4.85 (4.17-5.4) | 88 (89.8) | 0.483 |
| LV mass, g | 91.9 (76.1-108) | 1425 (84.7) | 101 (82.6-118) | 93 (94.9) | 0.001 |
| LV mass index, g/m2 | 46.7 (41.1-52.8) | 1425 (84.7) | 50.3 (43.0-57.5) | 93 (94.9) | 0.001 |
| LV global average wall thickness, mm | 5.92 (5.39-6.44) | 1423 (84.6) | 6.29 (5.55-6.87) | 93 (94.9) | <0.001 |
| **Cohort: type 2 diabetes** | | | | | |
| Ventricular rate, bpm | 66 (59-74) | 1683 (100) | 66 (57-75.8) | 98 (100) | 0.913 |
| QRS duration, ms | 86 (80-94) | 1683 (100) | 88 (82-98) | 98 (100) | 0.032 |
| QTc interval, ms | 424 (408-440) | 1683 (100) | 424 (402-441) | 98 (100) | 0.682 |
| T-wave offset, ms | 842 (820-864) | 1683 (100) | 839 (816-862) | 98 (100) | 0.325 |
| J-point amplitude (V3), mV | -0.015 (-0.044-0.019) | 1683 (100) | -0.005 (-0.044-0.028) | 98 (100) | 0.472 |
| T-wave amplitude (V3), mV | 0.332 (0.205-0.488) | 1667 (99) | 0.395 (0.191-0.501) | 98 (100) | 0.343 |
| J-point amplitude (aVL), mV | 0.004 (-0.015-0.029) | 1683 (100) | 0.0065 (-0.01-0.034) | 98 (100) | 0.395 |
| T-wave amplitude (aVL), mV | 0.097 (0.043-0.146) | 1582 (94) | 0.117 (0.022-0.185) | 87 (88.8) | 0.294 |
| Sokolow-Lyon index, mm | 19.1 (15.2-23.4) | 1526 (90.7) | 20.5 (16.2-25.3) | 86 (87.8) | 0.094 |
| LVEF, % | 56 (51-59) | 1412 (83.9) | 55 (50-59) | 89 (90.8) | 0.118 |
| LV ED volume, ml | 130 (109-155) | 1412 (83.9) | 136 (116-155) | 89 (90.8) | 0.296 |
| LV ES volume, ml | 58 (47-71) | 1412 (83.9) | 62 (51-74) | 89 (90.8) | 0.106 |
| LV stroke volume, ml | 72 (60-85) | 1412 (83.9) | 72 (58-83) | 89 (90.8) | 0.808 |
| Cardiac output, L/min | 4.7 (4-5.5) | 1412 (83.9) | 4.8 (4-5.4) | 89 (90.8) | 0.665 |
| LV mass, g | 90.9 (76.2-108) | 1426 (84.7) | 99.6 (85.8-107) | 92 (93.9) | 0.006 |
| LV mass index, g/m2 | 46.4 (41.2-52.1) | 1426 (84.7) | 48.7 (45.3-54.0) | 92 (93.9) | 0.001 |
| LV global average wall thickness, mm | 6.08 (5.56-6.6) | 1424 (84.6) | 6.28 (5.86-6.81) | 92 (93.9) | 0.006 |

**Table 3.** Logistic regression models used to quantify the association of selected biomarker predictors with incident cardiovascular disease (single binary outcome). Models are adjusted sequentially for different types of confounding factors. Socio-demographic factors include age, sex, ethnicity; lifestyle factors include body mass index (BMI), smoking; clinical factors include diastolic blood pressure, total cholesterol, triglycerides, C-reactive protein, anti-hypertensive medication and insulin.

|  | **No type 2 diabetes** | | | | | | | | | | | | |
| --- | --- | --- | --- | --- | --- | --- | --- | --- | --- | --- | --- | --- | --- |
|  | **Model 0**  Unadjusted | | | **Model 1**  Adjusted for socio-demographic factors | | | **Model 2**  Additionally adjusted for lifestyle factors | | | **Model 3**  Additionally adjusted for clinical factors | | | |
| **Predictor** | **N** | **Coefficient**  **(95% CI)** | **p-value** | **N** | **Coefficient**  **(95% CI)** | **p-value** | **N** | **Coefficient**  **(95% CI)** | **p-value** | **N** | **Coefficient**  **(95% CI)** | | **p-value** |
| QRS duration, ms | 1781 | 0.016  (0.004-0.027) | 0.007 | 1781 | 0.015  (0.002-0.027) | 0.019 | 1762 | 0.014  (0.002-0.026) | 0.022 | 1464 | 0.0137  (0-0.027) | 0.046 | |
| Sokolow-Lyon index, mm | 1650 | 0.049  (0.019-0.079) | 0.001 | 1650 | 0.049  (0.017-0.081) | 0.003 | 1633 | 0.051  (0.017-0.084) | 0.003 | 1364 | 0.051  (0.015-0.086) | 0.005 | |
| LV mass, g | 1518 | 0.017  (0.008-0.025) | <0.001 | 1518 | 0.0248  (0.014-0.036) | <0.001 | 1501 | 0.027  (0.015-0.039) | <0.001 | 1297 | 0.029  (0.017-0.042) | <0.001 | |
| LV mass index, g/m2 | 1518 | 0.047 (0.024-0.068) | <0.001 | 1518 | 0.058 (0.032-0.083) | <0.001 | 1501 | 0.054 (0.027-0.080) | <0.001 | 1297 | 0.059 (0.031-0.087) | <0.001 | |
| LV global average wall thickness, mm | 1516 | 0.508  (0.253-0.76) | <0.001 | 1516 | 0.623  (0.316-0.926) | <0.001 | 1499 | 0.707  (0.357-1.05) | <0.001 | 1295 | 0.677  (0.293-  1.06) | <0.001 | |

|  | **Type 2 diabetes** | | | | | | | | | | | |
| --- | --- | --- | --- | --- | --- | --- | --- | --- | --- | --- | --- | --- |
|  | **Model 0**  Unadjusted | | | **Model 1**  Adjusted for socio-demographic factors | | | **Model 2**  Additionally adjusted for lifestyle factors | | | **Model 3**  Additionally adjusted for clinical factors | | |
| **Predictor** | **N** | **Coefficient**  **(95% CI)** | **p-value** | **N** | **Coefficient**  **(95% CI)** | **p-value** | **N** | **Coefficient**  **(95% CI)** | **p-value** | **N** | **Coefficient**  **(95% CI)** | **p-value** |
| QRS duration, ms | 1781 | 0.0143  (0.002-0.026) | 0.020 | 1781 | 0.013  (0-0.025) | 0.045 | 1761 | 0.013  (0-0.025) | 0.042 | 1456 | 0.009  (-0.007-0.023) | 0.263 |
| Sokolow-Lyon index, mm | 1612 | 0.031  (-0.001-0.062) | 0.053 | 1612 | 0.030  (-0.003-0.062) | 0.071 | 1592 | 0.0363  (0.002-0.069) | 0.032 | 1335 | 0.0209  (-0.018-0.058) | 0.280 |
| LV mass, g | 1518 | 0.0103  (0.001-0.019) | 0.022 | 1518 | 0.0136  (0.003-0.024) | 0.013 | 1500 | 0.016  (0.003-0.027) | 0.011 | 1285 | 0.019  (0.006-0.032) | 0.003 |
| LV mass index, g/m2 | 1518 | 0.034 (0.009-0.056) | 0.006 | 1518 | 0.038 (0.011-0.064) | 0.005 | 1500 | 0.039 (0.012-0.066) | 0.004 | 1285 | 0.047 (0.017-0.075) | 0.001 |
| LV global average wall thickness, mm | 1516 | 0.354  (0.091-0.614) | 0.008 | 1516 | 0.413  (0.107-0.71) | 0.007 | 1498 | 0.457  (0.115-0.79) | 0.008 | 1283 | 0.561  (0.191-0.923) | 0.003 |

**Table 4.** Subgroup analysis: changes in biomarkers within the glycaemic spectrum. Multivariate multiple linear regression models to quantify the association of HbA1c with selected ECG and CMR-derived biomarkers. Models are adjusted sequentially for different types of confounding factors. Socio-demographic factors include age, sex, ethnicity; lifestyle factors include body mass index (BMI), smoking; clinical factors include diastolic blood pressure, total cholesterol, triglycerides, C-reactive protein, anti-hypertensive medication and insulin.

|  | **Model 0**  Unadjusted  **N = 1170** | | **Model 1**  Adjusted for socio-demographic factors  **N = 1170** | | **Model 2**  Additionally adjusted for lifestyle factors  **N = 1154** | | **Model 3**  Additionally adjusted for clinical factors  **N = 1014** | |
| --- | --- | --- | --- | --- | --- | --- | --- | --- |
| **Outcome** | **Coefficient**  **(95% CI)** | **p-value** | **Coefficient**  **(95% CI)** | **p-value** | **Coefficient**  **(95% CI)** | **p-value** | **Coefficient**  **(95% CI)** | **p-value** |
| **ECG** | | | | | | | | |
| Ventricular rate, bpm | 0.095  (0.037-0.153) | 0.001 | 0.094  (0.036-0.151) | 0.001 | 0.097  (0.040-0.154) | 0.001 | 0.12  (0.054-0.187) | 0.000 |
| QRS duration, ms | -0.020  (-0.088-0.047) | 0.557 | -0.009  (-0.073-0.056) | 0.79 | -0.0114  (-0.076-0.054) | 0.731 | 0.011  (-0.063-0.086) | 0.768 |
| QTc interval, ms | 0.012  (-0.102-0.126) | 0.835 | 0.004  (-0.105-0.114) | 0.937 | 0.009  (-0.100-0.118) | 0.871 | 0.026  (-0.106-0.157) | 0.702 |
| T-wave offset, ms | -0.26  (-0.425-(-0.096)) | 0.002 | -0.267  (-0.429-(-0.105)) | 0.001 | -0.279  (-0.441-(-0.116)) | 0.001 | -0.33  (-0.522-(-0.138)) | 0.001 |
| T-wave amplitude (V3), mV | 0.0001  (-0.001-0.001) | 0.859 | 0.0003  (-0.001-0.001) | 0.59 | 0.0002  (-0.001-0.001) | 0.642 | -0.001  (-0.002-0.000) | 0.19 |
| T-wave amplitude (aVL), mV | -0.001  (-0.001-0.000) | 0.012 | -0.001  (-0.001-0.000) | 0.013 | -0.001  (-0.001-0.000) | 0.017 | 0.000  (-0.001-0.000) | 0.151 |
| J-point amplitude (V3), mV | 0.000  (-0.0001-0.0003) | 0.547 | 0.0001  (-0.0001-0.0004) | 0.384 | 0.000  (-0.0001-0.0004) | 0.466 | 0.000  (-0.0003-0.0004) | 0.816 |
| J-point amplitude (aVL), mV | 0.000  (-0.0002-0.0002) | 0.869 | 0.000  (-0.0002-0.0002) | 0.817 | 0.000  (-0.0002-0.0002) | 0.925 | 0.000  (-0.0002-0.0002) | 0.784 |
| Sokolow-Lyon index, mm | 0.010  (-0.023-0.042) | 0.555 | 0.010  (-0.022-0.042) | 0.558 | 0.008  (-0.024-0.039) | 0.638 | 0.006  (-0.031-0.044) | 0.736 |
| **CMR** | | | | | | | | |
| LV ED volume, ml | -0.363  (-0.837-0.111) | 0.133 | -0.347  (-0.814-0.121) | 0.146 | -0.323  (-0.797-0.151) | 0.182 | -0.256  (-0.852-0.34) | 0.4 |
| LV stroke volume, ml | -0.135  (-0.256-(-0.014)) | 0.029 | -0.132  (-0.247-(-0.018)) | 0.023 | -0.122  (-0.237-(-0.007)) | 0.038 | -0.104  (-0.243-0.036) | 0.145 |
| LV global average wall thickness, mm | -0.002  (-0.006-0.002) | 0.378 | -0.001  (-0.005-0.002) | 0.408 | -0.0003  (-0.003-0.003) | 0.86 | 0.001  (-0.003-0.004) | 0.607 |

**Table 5.** Subgroup analysis: females versus males. IQR: inter-quartile range, ECG: electrocardiogram, CMR: cardiac magnetic resonance, LV: left ventricular, EF: ejection fraction, ED: end-diastolic, ES: end-systolic. All continuous variables are distributed non-normally and compared using the Mann-Whitney U-test.

|  | **No type 2 diabetes** | | **Type 2 diabetes** | |  |
| --- | --- | --- | --- | --- | --- |
|  | **Median (IQR)** | **N (%)** | **Median (IQR)** | **N (%)** | **p-value** |
| **Cohort: females** | | | | | |
| Ventricular rate, bpm | 62 (57-70) | 648 (100) | 67 (60-74) | 648 (100) | <0.001 |
| QRS duration, ms | 82 (76-90) | 648 (100) | 82 (76-88) | 648 (100) | 0.048 |
| QTc interval, ms | 429 (413-443) | 648 (100) | 433 (417-447) | 648 (100) | 0.001 |
| T-wave offset, ms | 858 (838-876) | 648 (100) | 850 (826-870) | 648 (100) | <0.001 |
| J-point amplitude (V3), mV | -0.03 (-0.054-0) | 648 (100) | -0.025 (-0.054-0.004) | 648 (100) | 0.208 |
| T-wave amplitude (V3), mV | 0.263 (0.156-0.375) | 638 (98.5) | 0.239 (0.141-0.341) | 635 (98) | 0.040 |
| J-point amplitude (aVL), mV | 0.004 (-0.015-0.029) | 648 (100) | 0.004 (-0.015-0.029) | 648 (100) | 0.381 |
| T-wave amplitude (aVL), mV | 0.097 (0.043-0.156) | 615 (94.9) | 0.087 (0.007-0.132) | 600 (92.6) | 0.006 |
| Sokolow-Lyon index, mm | 18.1 (14.8-22.1) | 609 (94) | 17.4 (14.2-21.5) | 584 (90.1) | 0.019 |
| LVEF, % | 57 (54-61) | 564 (87) | 57 (53-61) | 551 (85) | 0.526 |
| LV ED volume, ml | 120 (104-138) | 564 (87) | 113 (97-130) | 551 (85) | <0.001 |
| LV ES volume, ml | 51 (43-60) | 564 (87) | 48 (41-57.5) | 551 (85) | <0.001 |
| LV stroke volume, ml | 68 (58-79) | 564 (87) | 64 (54-74) | 551 (85) | <0.001 |
| Cardiac output, L/min | 4.2 (3.7-4.8) | 564 (87) | 4.3 (3.6-4.9) | 551 (85) | 0.852 |
| LV mass, g | 72.8 (63.9-81.2) | 562 (86.7) | 73.2 (65.4-84.2) | 556 (85.8) | 0.062 |
| LV mass index, g/m2 | 40.1 (36.9-43.6) | 562 (86.7) | 41.1 (36.4-45.2) | 556 (85.8) | 0.089 |
| LV global average wall thickness, mm | 5.31 (4.98-5.67) | 561 (86.6) | 5.55 (5.17-5.94) | 556 (85.8) | <0.001 |
| **Cohort: males** | | | | | |
| Ventricular rate, bpm | 60 (54-67) | 1133 (100) | 65 (58-73) | 1133 (100) | <0.001 |
| QRS duration, ms | 90 (84-98) | 1133 (100) | 90 (84-98) | 1133 (100) | 0.018 |
| QTc interval, ms | 416 (401-431) | 1133 (100) | 419 (404-434) | 1133 (100) | 0.004 |
| T-wave offset, ms | 850 (832-872) | 1133 (100) | 838 (814-860) | 1133 (100) | <0.001 |
| J-point amplitude (V3), mV | -0.005 (-0.035-0.034) | 1133 (100) | -0.005 (-0.035-0.034) | 1133 (100) | 0.532 |
| T-wave amplitude (V3), mV | 0.434 (0.302-0.61) | 1128 (99.6) | 0.395 (0.258-0.55) | 1130 (99.7) | <0.001 |
| J-point amplitude (aVL), mV | 0.009 (-0.01-0.034) | 1133 (100) | 0.009 (-0.015-0.029) | 1133 (100) | 0.017 |
| T-wave amplitude (aVL), mV | 0.117 (0.063-0.17) | 1103 (97.4) | 0.102 (0.048-0.156) | 1069 (94.4) | <0.001 |
| Sokolow-Lyon index, mm | 21.4 (17.2-25.9) | 1041 (91.9) | 20.4 (16-24.5) | 1028 (90.7) | <0.001 |
| LVEF, % | 55 (51-59) | 969 (85.5) | 54 (50-58) | 950 (83.8) | 0.072 |
| LV ED volume, ml | 152 (134-173) | 969 (85.5) | 142 (122-165) | 950 (83.8) | <0.001 |
| LV ES volume, ml | 68 (58-80) | 969 (85.5) | 65 (53-77) | 950 (83.8) | <0.001 |
| LV stroke volume, ml | 83 (72-96) | 969 (85.5) | 77 (64-90) | 950 (83.8) | <0.001 |
| Cardiac output, L/min | 5 (4.4-5.8) | 969 (85.5) | 5 (4.2-5.8) | 950 (83.8) | 0.340 |
| LV mass, g | 103 (92.6-114) | 956 (84.4) | 103 (89.7-115) | 962 (84.9) | 0.158 |
| LV mass index, g/m2 | 51.1 (46.3-55.5) | 956 (84.8) | 50.2 (45.3-54.9) | 962 (84.9) | 0.005 |
| LV global average wall thickness, mm | 6.26 (5.86-6.73) | 955 (84.3) | 6.37 (5.97-6.84) | 960 (84.7) | <0.001 |

**Table 6.** Subgroup analysis: white versus non-white participants. IQR: inter-quartile range, ECG: electrocardiogram, CMR: cardiac magnetic resonance, LV: left ventricular, EF: ejection fraction, ED: end-diastolic, ES: end-systolic. All continuous variables are distributed non-normally and compared using the Mann-Whitney U-test.

|  | **No type 2 diabetes** | | **Type 2 diabetes** | |  |
| --- | --- | --- | --- | --- | --- |
|  | **Median (IQR)** | **N (%)** | **Median (IQR)** | **N (%)** | **p-value** |
| **Cohort: non-white ethnic background** | | | | | |
| Ventricular rate, bpm | 59.5 (56.5-66) | 48 (100) | 67 (59-76) | 141 (100) | <0.001 |
| QRS duration, ms | 84 (80-90.5) | 48 (100) | 84 (78-92) | 141 (100) | 0.871 |
| QTc interval, ms | 415 (397-432) | 48 (100) | 420 (406-440) | 141 (100) | 0.141 |
| T-wave offset, ms | 851 (833-867) | 48 (100) | 836 (812-860) | 141 (100) | 0.004 |
| J-point amplitude (V3), mV | -0.008 (-0.045-0.043) | 48 (100) | 0 (-0.04-0.048) | 141 (100) | 0.406 |
| T-wave amplitude (V3), mV | 0.375 (0.229-0.556) | 47 (97.9) | 0.312 (0.194-0.463) | 140 (99.3) | 0.188 |
| J-point amplitude (aVL), mV | 0.024 (0.003-0.048) | 48 (100) | 0.009 (-0.015-0.034) | 141 (100) | 0.027 |
| T-wave amplitude (aVL), mV | 0.117 (0.085-0.188) | 47 (97.9) | 0.112 (0.063-0.17) | 135 (95.7) | 0.130 |
| Sokolow-Lyon index, mm | 20.9 (17.4-25.9) | 40 (83.3) | 20.4 (15.5-25.7) | 124 (87.9) | 0.368 |
| LVEF, % | 56 (51-60) | 38 (79.2) | 56 (53-60) | 112 (79.4) | 0.898 |
| LV ED volume, ml | 124 (115-146) | 38 (79.2) | 119 (97.8-139) | 112 (79.4) | 0.146 |
| LV ES volume, ml | 56.5 (47-71) | 38 (79.2) | 51 (43-66) | 112 (79.4) | 0.156 |
| LV stroke volume, ml | 68.5 (62-83.5) | 38 (79.2) | 66 (54-78) | 112 (79.4) | 0.143 |
| Cardiac output, L/min | 4.45 (3.73-5.08) | 38 (79.2) | 4.5 (3.6-5.3) | 112 (79.4) | 0.782 |
| LV mass, g | 93.3 (81.2-103) | 38 (79.2) | 86.5 (70.3-103) | 110 (78) | 0.285 |
| LV mass index, g/m2 | 46.9 (42.4-52.7) | 38 (79.2) | 45.8 (39.8-52.5) | 110 (78) | 0.189 |
| LV global average wall thickness, mm | 6.26 (5.51-6.68) | 38 (79.2) | 6.01 (5.51-6.55) | 110 (78) | 0.484 |
| **Cohort: white ethnic background** | | | | | |
| Ventricular rate, bpm | 61 (55-68) | 1733 (100) | 66 (59-74) | 1640 (100) | <0.001 |
| QRS duration, ms | 88 (82-96) | 1733 (100) | 86 (80-96) | 1640 (100) | 0.013 |
| QTc interval, ms | 421 (405-436) | 1733 (100) | 424 (408-440) | 1640 (100) | <0.001 |
| T-wave offset, ms | 854 (834-874) | 1733 (100) | 842 (820-864) | 1640 (100) | <0.001 |
| J-point amplitude (V3), mV | -0.015 (-0.044-0.019) | 1733 (100) | -0.015 (-0.044-0.019) | 1640 (100) | 0.699 |
| T-wave amplitude (V3), mV | 0.366 (0.229-0.537) | 1719 (99.2) | 0.332 (0.205-0.488) | 1625 (99.1) | <0.001 |
| J-point amplitude (aVL), mV | 0.009 (-0.01-0.029) | 1733 (100) | 0.004 (-0.015-0.029) | 1640 (100) | 0.021 |
| T-wave amplitude (aVL), mV | 0.107 (0.053-0.166) | 1671 (96.4) | 0.097 (0.039-0.146) | 1534 (93.5) | <0.001 |
| Sokolow-Lyon index, mm | 20.1 (16.2-24.7) | 1610 (92.9) | 19.1 (15.2-23.4) | 1488 (90.7) | <0.001 |
| LVEF, % | 56 (52-59) | 1495 (86.3) | 55 (51-59) | 1389 (84.7) | 0.041 |
| LV ED volume, ml | 141 (119-163) | 1495 (86.3) | 131 (110-156) | 1389 (84.7) | <0.001 |
| LV ES volume, ml | 61 (51-74) | 1495 (86.3) | 58 (48-72) | 1389 (84.7) | <0.001 |
| LV stroke volume, ml | 78 (65-90) | 1495 (86.3) | 72 (60-85) | 1389 (84.7) | <0.001 |
| Cardiac output, L/min | 4.7 (4.1-5.6) | 1495 (86.3) | 4.7 (4-5.5) | 1389 (84.7) | 0.730 |
| LV mass, g | 92.5 (76.3-109) | 1480 (85.4) | 91.6 (77.2-108) | 1408 (85.9) | 0.555 |
| LV mass index, g/m2 | 46.9 (41.1-53.0) | 1480 (85.4) | 46.8 (41.4-52.3) | 1408 (85.9) | 0.70 |
| LV global average wall thickness, mm | 5.92 (5.4-6.46) | 1478 (85.3) | 6.09 (5.6-6.62) | 1406 (85.7) | <0.001 |
